# BMP-2 and VEGF-A modRNAs in collagen scaffold synergistically drive bone repair through osteogenic and angiogenic pathways

**DOI:** 10.1038/s42003-020-01606-9

**Published:** 2021-01-19

**Authors:** Yingnan Geng, Huichuan Duan, Liang Xu, Nevin Witman, Bingqian Yan, Zheyuan Yu, Huijing Wang, Yao Tan, Liqin Lin, Dong Li, Shanshan Bai, Regina Fritsche-Danielson, Jie Yuan, Kenneth Chien, Min Wei, Wei Fu

**Affiliations:** 1 Department of Plastic and Reconstructive Surgery, Shanghai 9th People’s Hospital, School of Medicine, Shanghai Jiao Tong University, 639 Zhi Zao Ju Road, 200011 Shanghai, China; 2 Department of Medicine, Karolinska Institutet, Stockholm, Sweden; 3 Department of Cell and Molecular Biology, Karolinska Institutet, Stockholm, Sweden; 4 Institute of Pediatric Translational Medicine, Shanghai Children’s Medical Center, School of Medicine, Shanghai Jiao Tong University, 1678 Dong Fang Road, 200127 Shanghai, China; 5 Department of Pediatric Cardiothoracic Surgery, Shanghai Children’s Medical Center, School of Medicine, Shanghai Jiao Tong University, 1678 Dong Fang Road, 200127 Shanghai, China; 6 Research and Early Clinical Development, Cardiovascular, Renal and Metabolism, Biopharmaceuticals R&D, AstraZeneca, Pepparedsleden 1, 43183 Gothenburg, Sweden; 7 Shanghai Key Laboratory of Tissue Engineering, Shanghai 9th People’s Hospital, School of Medicine, Shanghai Jiao Tong University, 200011 Shanghai, China

**Keywords:** Stem-cell differentiation, Translational research, Tissue engineering, Bone

## Abstract

Bone has a remarkable potential for self-healing and repair, yet several injury types are non-healing even after surgical or non-surgical treatment. Regenerative therapies that induce bone repair or improve the rate of recovery are being intensely investigated. Here, we probed the potential of bone marrow stem cells (BMSCs) engineered with chemically modified mRNAs (modRNA) encoding the hBMP-2 and VEGF-A gene to therapeutically heal bone. Induction of osteogenesis from modRNA-treated BMSCs was confirmed by expression profiles of osteogenic related markers and the presence of mineralization deposits. To test for therapeutic efficacy, a collagen scaffold inoculated with modRNA-treated BMSCs was explored in an in vivo skull defect model. We show that hBMP-2 and VEGF-A modRNAs synergistically drive osteogenic and angiogenic programs resulting in superior healing properties. This study exploits chemically modified mRNAs, together with biomaterials, as a potential approach for the clinical treatment of bone injury and defects.

## Abstract (web summary)

Geng et al. evaluate bone marrow stem cell (BMSC)-based system to deliver modified RNAs of BMP-2 and VEGF to enhance bone regeneration. They test its therapeutic efficacy in vivo on a rat skull defect model by inoculating these BMSCs in a collagen scaffold. This construct synergistically drives osteogenic and angiogenic pathways and can be a new approach for clinical treatment of bone injuries and defects.

## Introduction

Several non-healing bone defects arise from severe trauma, nonunion fractures, tumor resection or craniomaxillofacial surgery. Such impairments occur despite the bones’ natural endogenous repair and regeneration capacity. In order to achieve entire healing a variety of means have been explored, which include bone grafts (autograft or allograft), mesenchymal stem/stromal cells (MSCs), tissue-engineered bone grafts (TEBG) or a combination of biomaterials^1,2^ and growth factors, such as human bone morphogenetic protein 2 (BMP-2)^3,4^. Indeed, the over expression of BMP-2, a well-known growth differentiation factor, can stimulate osteoblasts from an MSC population both in vitro and in vivo^5^. BMP-2 has been extensively studied in the research of bone regeneration^6^ and in particular as an applied treatment through the delivery of a biodegradable collagen sponge, which showed effective results in the treatment of patients with bone fractures, as well as oral and maxillofacial osseous defects^7,8^. On the other hand, human vascular endothelial growth factor (VEGF-A) plays a key role in early stages of osteogenesis and has considerable functions in mechanisms underlying skeletal growth and repair^9^. Taken together, human BMP-2 and VEGF-A are two well-known factors that are key developmental and reparative regulators of bone growth^10,11^.

Conventional recombinant protein therapies have limitations in terms of technological limitations due to the short half-life period associated with these growth factors^12,13^ (VEGF half-life is approximately 13 min long, and BMP-2 is 7 to 16 min). Clinically, these growth factors are administrated at supraphysiological dosages in order to achieve successful therapeutic efficacy. The administration of such high doses of recombinant proteins is also required to overcome the low bioavailability in vivo^14^, which often leads to adverse effects^15,16^. Recently, gene-based therapies in the form of transgenes have proven to be effective for bone regeneration^17^. Consequently, gene therapy represents a potential therapeutic option for bone healing^18^. However, gene-based therapies for clinical medicine continue to face technical challenges and safety risks for patients, including induction of DNA mutations or an inflammatory response to the delivery system causing a surge of activated immune cells. Therefore, new bone regeneration strategies that are met with patient safety and are cost-efficient will be in great demand in the field of medicine and surgery.

Unlike gene therapy, modified mRNAs deliver a transient “burst” of therapeutic protein expression while alleviating immune responses often associated with the delivery of exogenous biological entities^19^. Towards this goal, chemically modified mRNAs have begun to vastly emerge as a promising therapeutic platform, the technology of which has been showcased in several pre-clinical/clinical studies^20–22^. Previous reports using modified mRNAs have relied on lipid nanoparticles (LNPs), a known cytotoxic compound for successful delivery^20,23–25^. Our team has recently reported a method for enhancing mRNA delivery and subsequent in vivo produced protein using a cell-based technology system^26^, which could circumvent issues related to LNP cytotoxicity. Moreover, the utilization of BMP modRNA (modBMP2) as a non-integrating, non-viral, mRNA approach for effective treatment of bone defects has recently gained substantial merit over gene-based therapies^27–29^. We therefore set out to take the technology one step further by employing a combinatorial approach that unites the mRNA technology platform with cell therapy and biomaterials for bone repair.

Here we investigate the osteogenic and bone regeneration capacity of mRNA-engineered BMSCs. We reveal that the co-expression of modBMP2 with VEGF-A modRNA (modVEGF) induced ALP activity and more effectively promote the formation of calcium nodules when compared to administration of single treatment groups and recombinant proteins. Furthermore, BMSCs secreting a combination of BMP2 and VEGF-A proteins more efficiently stimulate accelerated bone healing and regeneration by enhancing osteogenesis through the simultaneous stimulation of angiogenesis in an in vivo rat calvarial defect model. These results provide insight to a safe, efficient and cost-effective approach for bone tissue regeneration that abridges mRNA and cell-based therapies.

## Results

### Synthetic chemically modified mRNAs are efficiently taken up in BMSCs

**Fig. 1:**
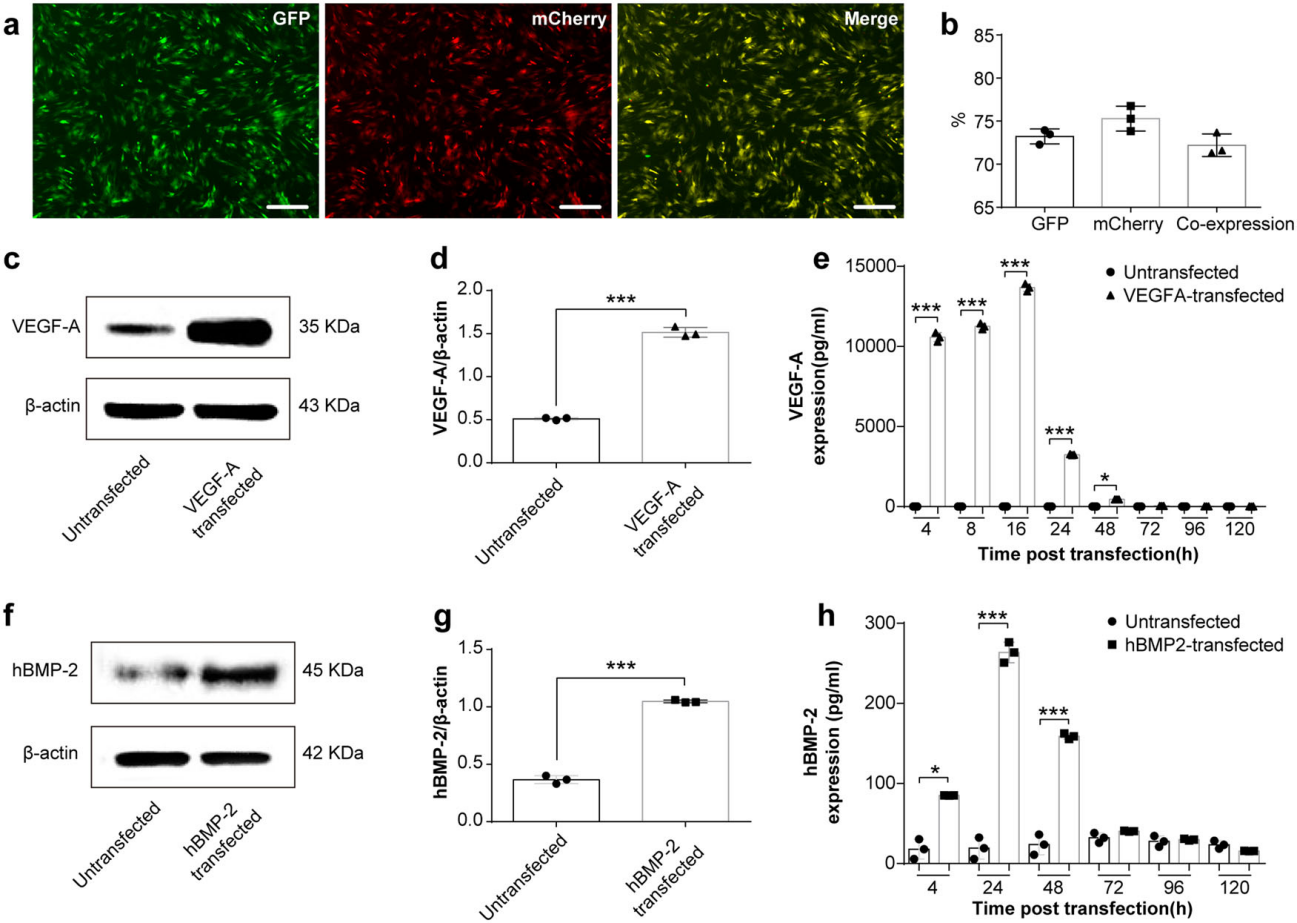
Efficiency and protein kinetics of modRNA transfection in BMSCs. **a** Representative fluorescence microscopy images of BMSCs taken 24 h after GFP and mCherry modRNA co-transfection. The scale bars represent 250 μm. **b** Flow data analysis depicted as a histogram revealing efficiency of co-transfections. (**c**–**d**, **f**, **g**) Comparison of VEGF-A (**c**–**d**) or BMP-2 (**f**, **g**) protein expression between naïve BMSCs and modRNA-transfected BMSCs at 24 h post transfection. **e**, **h** VEGF-A (**e**) or hBMP-2 (**h**) protein expression kinetics at indicated times following modRNA transfections. Data results are expressed as mean ± SD, *n* = 3, *p < 0.05, **p < 0.01, ***p < 0.001. (*p < 0.05) Indicate difference on secreted hBMP-2 or VEGF-A between untreated and transfected cells.

Firstly, four different mRNA molecules encoding GFP, mCherry, BMP-2, and VEGF-A were modified by the full replacement of uridine-5’-triphosphate with N^1^-methylpseudo-uridine-5’-triphosphate (m1ψ), as previously described^26^. As our goal was to co-transfect and simultaneously secrete two growth factors from BMSCs, we performed single and co-administered transfections of GFP and mCherry reporters. The expression kinetics of the single administered and co-transfected modified mRNAs in BMSCs were analyzed and compared at 24 h post-transfection (hpt) using fluorescence microscopy and photomicrographic images (Fig. 1a). Flow cytometry analysis revealed that single transfections yielded >90% expression in BMSCs 24 hpt (Fig. S1a–c). When modRNAs were co-administered, the total proportion of cells expressing either one or both of the modified mRNA reporters were between 70–75%, Fig. 1a, b. The calculated modRNA dosage used in all our studies equates to 10 pg per cell in total in all groups.

In order to confirm that the LNP transfections have no negative effect on cellular health and function, we evaluated the proliferation of modGFP transfected-BMSCs over 48 h using a CCK-8 assay^30^. As shown in Fig. S1d, the proliferation for modRNA transfected BMSCs was similar to the untransfected group, suggesting that modRNA-polyplexes have no influence on cell proliferation, a vital indicator of cell health^31^.

### modRNA transfected BMSCs express and secrete high levels of BMP-2 and VEGF-A protein

In order to confirm successful mRNA uptake, RNA was isolated from BMSCs that had been transfected with modBMP2 and modVEGF-A at 24 hpt. Consequently, mRNA transcript levels were quantified by qRT-PCR. Compared to untransfected BMSCs, the modRNA transfections resulted in higher levels of mRNA inside the cells (***p < 0.001), (Supplementary Fig. 1e, f).

To evaluate BMP2 and VEGF-A target protein expression levels after transfection, we quantified protein levels found in cell culture supernatants 24 hpt and found an increase above basal levels compared to untransfected groups (Fig. 1c–d, f, g and Supplementary Fig. 9) respectively (***p < 0.001). Next, in order to capture and quantify secreted protein levels of BMP2 and VEGF-A over the long-term interim, cell culture supernatants were collected at different time points following single-administered transfections. Quantification of BMP2 and VEGF-A protein using ELISA (R&D Systems, MN, USA) revealed increases in levels of cumulative protein secretion for at least the first 2 days in both groups (***p < 0.001), (Supplementary Fig. 1g, h). According to a time course evaluation, maximum VEGF-A expression was observed 16 hpt, while maximum BMP2 expression was observed at 24 hpt, (Fig. 1e, h). Similarly, following co-transfections of both BMP2 and VEGFA modRNA, protein quantification revealed increases in levels of respective secreted proteins over the course of 2 days with both protein levels peaking around 24 hpt (***p < 0.001), (Supplementary Fig. 1i, j).

### Osteogenic differentiation of BMSCs is enhanced by modBMP2 and modVEGF-A transfection in vitro

**Fig. 2:**
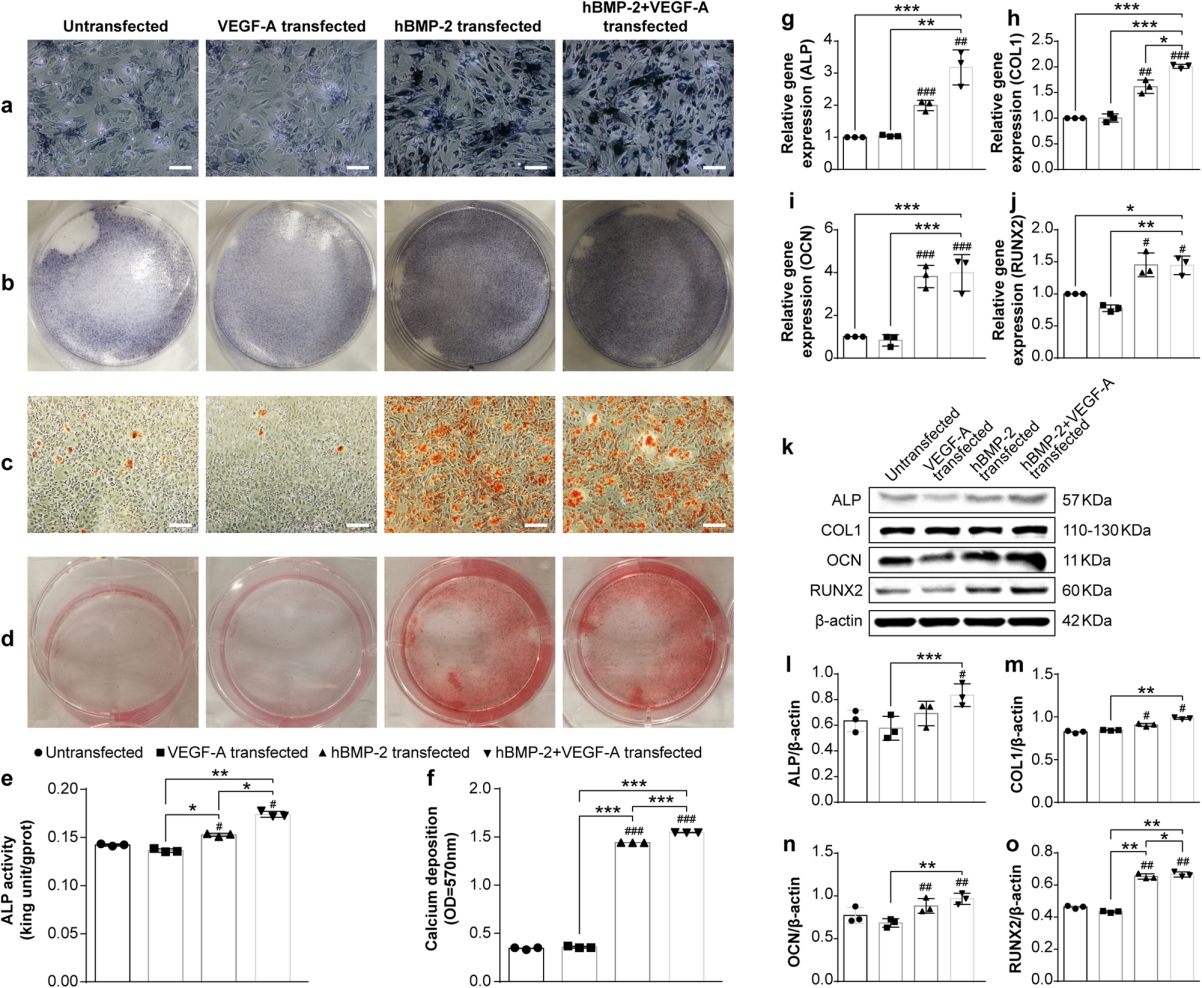
Molecular and cellular detection of in vitro osteogenesis following modRNA transfection in BMSCs. **a** Alkaline phosphatase (ALP) staining of BMSCs at 7 days post-transfection in different modRNA treatment groups. The scale bars represent 125 μm. **b** Gross appearance of ALP staining. **c** Detection of mineralized matrix using alizarin red staining 14 days post-transfection in indicated modRNA treatment groups. **d** Gross appearance of alizarin red staining 14 days post transfection. The scale bars represent 250 μm. **e**, **f** Quantification of ALP activity (**e**) 7 days post-transfection and quantification of alizarin red staining (**f**) 14 days post-transfection. Significant differences between untreated and modRNA-transfected cells are indicated by (*p < 0.05, **p < 0.01, ***p < 0.001). *N* = 6, one-way ANOVA test. **g**–**j** Fold increase in gene expression of (**g**) ALP, (**h**) Collagen Type I (COL 1), (**i**) Osteocalcin (OCN), and (**j**) Runt-related transcription factor 2 (RUNX2) at 7 days post-transfection determined by qRT-PCR analysis. **k**–**o** Representative western blot assessment (**k**) and quantification of protein expression of ALP (**l**), COL1 (**m**), OCN (**n**), and RUNX2 (**o**) following transfection of indicated modRNA treatments. Hash (#) indicate difference between non-transfection and transfection groups. Astersisk (*) indicate difference among different modRNA transfection groups. Results are normalized to the housekeeping gene (rat β-actin) and to the untreated cells. Values are shown as mean ± SD, ^#/^*p < 0.05, ^##/^**p < 0.01, ^###/^***p < 0.001. *N* = 6, One-way ANOVA followed by Tukey’s multiple comparison.

To gain insight into the osteogenic potency of BMSCs engineered with modBMP2 and modVEGF we explored a series of in vitro assays. First, we explored the capability of the single and co-transfected groups to increase alkaline phosphatase (ALP) and calcium deposition in conjunction with standard differentiation protocols that contain dexamethasone, a known mineralization agent^32^. ALP is an early indicator of osteogenic differentiation, therefore increased ALP activity in our modRNA-transfected BMSCs would be a desirable feature. We assessed BMSCs for ALP activity following transfection of modBMP2 alone, modVEGF alone, or as a combinatorial treatment together, 1:1 modBMP2+modVEGF-A (1B1V). ALP expression was observed in the modBMP2 and 1B1V modRNA treatment groups as early as 7 days post transfection (Fig. 2a, b), but not in the modVEGF-A transfected group. In addition, quantitative analysis of ALP activity through a colorimetric assay revealed a significant increase in ALP levels stemming from the modBMP group and the 1B1V group in contrast to untransfected BMSCs (Fig. 2e, *p < 0.05, **p < 0.01).

Calcium deposition is a late stage marker for osteogenic differentiation^33^. We next tested if our BMSCs could induce mineralization as a result of modBMP2 and modVEGF-A transfections. Indeed, microscopic analysis of alizarin red staining at 14dpt revealed an increased number of calcium nodules in the modBMP2 group and a more pronounced effect in the 1B1V modRNA treatment group over the control group (Fig. 2c, d). Mineralization did not appear to occur in the modVEGF-A treatment group as alizarin red staining levels were consistent with levels seen in the control group. Quantitative assessment of the alizarin red staining (Fig. 2f) confirmed these results (*p* < 0.001).

Next, the expression of osteogenic related genes was quantified at 7 days post osteogenic induction (dpoi) by qRT-PCR, as shown in Fig. 2g–j. Expression of ALP, COL1, and OCN were all increased by 1B1V modRNA treatment (*p* < 0.001, *N* = 3) as compared to single treatment groups and controls. A similar effect was seen with Runx2, but to a lesser extent (*P* < 0.05, *N* = 3). Subsequently, western blot analysis was performed to evaluate quantitative changes in protein levels of these osteogenic-related genes following modRNA treatment. BMSCs treated with modBMP2 exerted increased protein levels of osteogenic genes over controls. Further, as seen with mRNA/gene expression levels the modVEGF-A treatment group did not elicit increased protein levels of major osteogenic genes, however the protein levels of ALP, COL1, OCN, and Runx2 were further increased in BMSCs treated with modBMP2 alone and increased in the 1B1V treatment group (*P* < 0.05, *N* = 3), as shown in Fig. 2k–o and Supplementary Fig. 10.

In addition, we performed a ratio optimization test in the absence of dexamethasone where we also incorporated recombinant human proteins as controls (Fig. S2). Interestingly, we discovered statistical improvement in ALP activity and calcium deposition in both the 1B1V, as well as the 3:1 modBMP2 + modVEGF (3B1V) treatment groups over the controls and recombinant protein groups (Fig. S2a–f). In addition, the modRNA treatment groups performed equally as well or better than the recombinant protein groups when it came to osteogenic gene expression and stimulation (Supplementary Fig. 2g–o, Supplementary Fig. 12 and Supplementary Fig. 14). A 1:3 ratio of modBMP2 + modVEGF (1B3V) was less capable of activating osteogenic gene networks (Supplementary Fig. 2a–o).

The potential for modVEGF-A to induce angiogenesis by the assessment of an endothelial tube formation assay was explored, shown in Supplementary Fig. 3. The total branching length and number of nodes in the modVEGF-A transfected group were higher than that of those found in the untransfected group (*P* < 0.05, *N* = 3). The representative images of the tube formation assay revealed that modVEGF-A treated HUVEC cells increased branch length and the numbers of endothelial tubes compared to those in normal culture medium. Together these studies provide credence for a functionally active therapeutic cell capable of driving osteogenic induction (Fig. 2) together with angiogenesis (Supplementary Fig. 3) following the transfection of chemically modified mRNAs.

### In vivo transplantation of modified mRNA-treated BMSCs enhance bone repair

To investigate the in vivo protein expression kinetics resulting from modRNA-treated BMSCs, bioluminescence imaging was performed. BMSCs were transfected with a luciferase modRNA construct (as previously designed)^26^ and at 24 h time intervals following implantation to the skull bone, animals were sedated and non-invasively imaged. As illustrated in Fig. S4 luciferase expression was detected and peaked after 1 day with an average value of 1.5 × 10^5^ photons/sec/cm^2^/sr. As expected, signal intensities declined gradually over the course of 3 days (Supplementary Fig. 4) but maintained a signal over baseline (remaining activity of 6.6 × 10^2^ photons/sec/cm^2^/sr) on day 4 post-treatment. However, after 4 days luciferase activity dropped to baseline values.

**Fig. 3:**
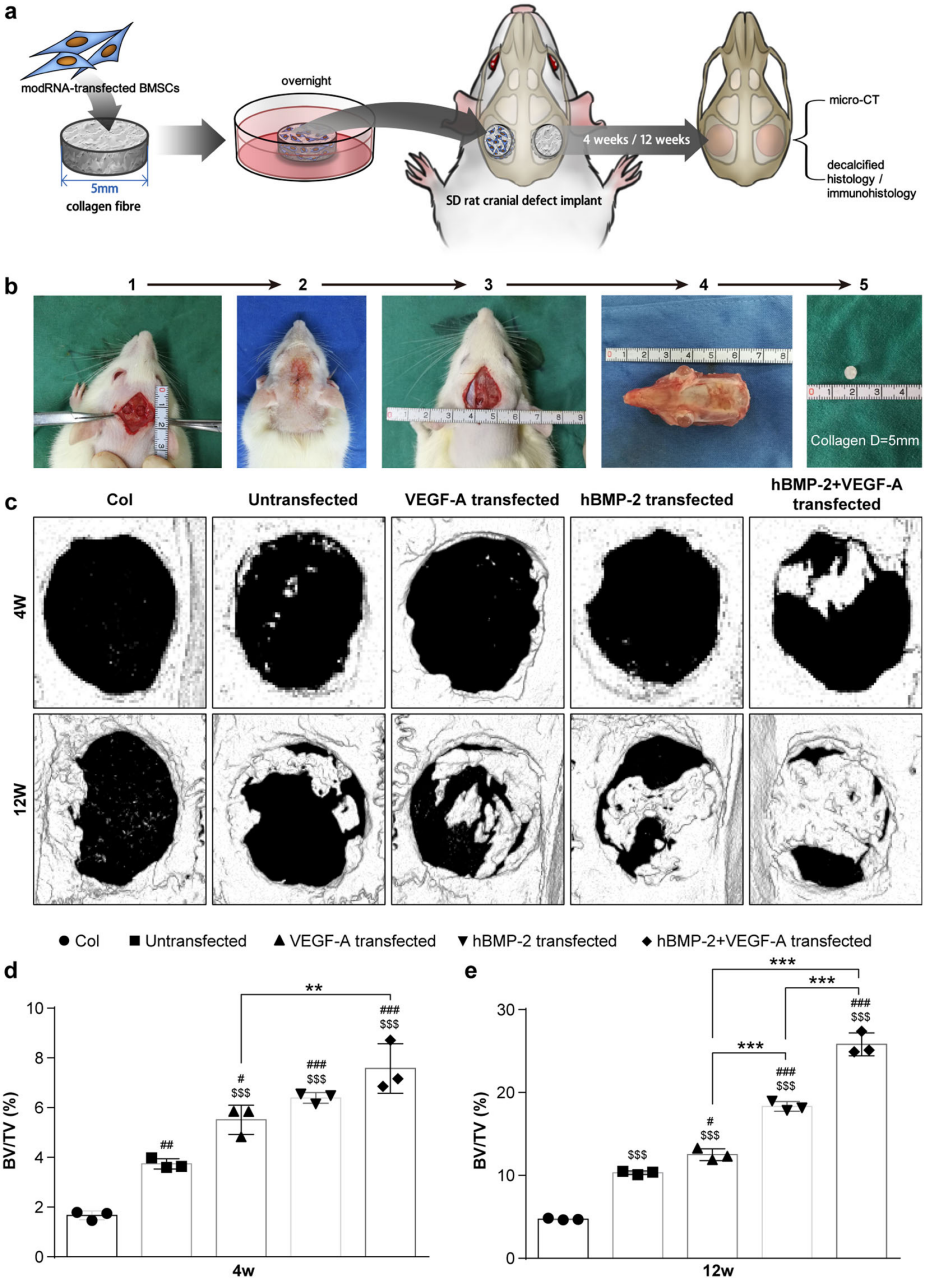
modRNA treated BMSCs combined with biomaterials enhance bone healing in rat cranial defect model. (a-) Schematic of experimental design (**a**) and cranial defect surgery process (**b**). **c** Representative images reconstructed from 3D μCT scans reveals bone tissue regeneration at 4 and 12 wks after treatment. **d**, **e** Quantified bone volume fraction (BV/TV) of regenerated bone at 4 wks (**d**) and 12 wks (**e**) post-implantation. Differences between modRNA treatments and control group were assessed by one-way ANOVA followed by Tukey’s multiple comparison. Dollar ($) indicate difference between acellular collagen scaffold group and other four groups. Hash (#) indicate difference between non-transfected and transfected groups. Asterisk (*) indicate difference among modRNA transfection groups. (Values are expressed as mean ± SD, ^$/#/^*p < 0.05, ^$$/##/^**p < 0.01, ^$$$/###/^***p < 0.001).

Next, in order to assess for efficacy, BMSCs were implanted in rats, which underwent cranial defect surgery as previously described^34^. A collagen fiber matrix was loaded with modRNA-transfected BMSCs and surgically implanted at the orthotopic site (Fig. 3a, b). The rats were sacrificed at 4 and 12 weeks (wks) post-operation, where the degree of bone healing was evaluated through histology and micro-computed tomography (µCT) scans to calculate percent bone volume (BV/TV).

According to µCT scans, we found the combinatorial treatment of 1B1V modRNA was most capable of generating new bone tissue deposition that fractionally filled the defect space at 4 and 12 wks following transplantation (Fig. 3c). Quantitative analysis of μCT scans revealed the 1B1V modRNA treatment group increased neo-bone formation over all other groups both at 4 and 12 wks (*P* < 0.01) and gave rise to higher BV/TV ratios than single treatments or control groups (Fig. 3d, e). The BV/TV was 2.7-fold higher in defects treated with the 1B1V modRNA-loaded collagen scaffold when compared to the untreated group, 2.45-fold higher than the modVEGF-A treated group, and 1.59-fold higher than the modBMP2 treated group at 12 wks, respectively.

### Histological evaluation and immunohistology analysis

**Fig. 4:**
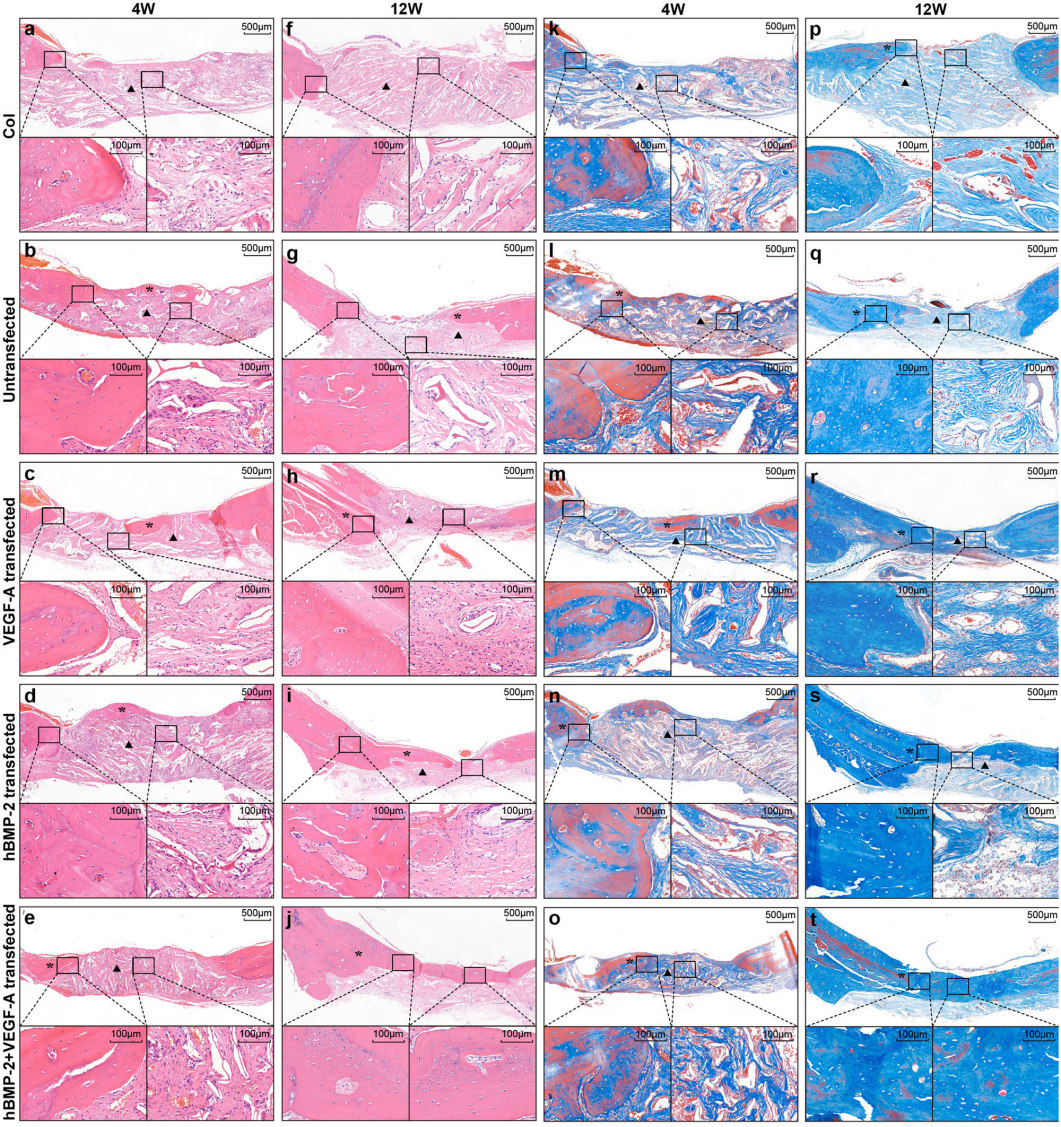
Histological evaluation of bone defects and repair following injury and scaffold implantation. **a**–**j** Hematoxylin and eosin staining at the location of the cranial defect 4weeks (**a**–**e**) and 12 weeks (**f**–**j**) post-implantation. Note less neo-bone formation in the control and single treatment groups (asterisks). **k**–**t** Masons trichrome staining at the location of the cranial defect 4 wks (**k**–**o**) and 12 wks (**p**–**t**) post-treatment. At 4weeks post-treatment increased levels of bone-forming osteoids were detected in modRNA treatment groups, as indicated by blue staining (**n**, **o**). At 12weeks post-treatment there is more mineralized bone in the modRNA treatment groups as indicated by blue staining (**s**, **t**). The most different level of bone repair was found in the group of scaffolds embedded with 1B1V transfected BMSCs. (Note: in all images the asterisks represent callus tissue; the arrowheads represent fibrous connective tissue).

Histological stainings were assessed to further evaluate bone regeneration from the modRNA-transfected collagen scaffolds at week 4 and 12 wks post-surgery and transplantation. Distinct patterns of neobone formation were observed in the orthotopic environment following the administration of different modRNA-transfected BMSC scaffolds. The 1B1V modRNA transfection group appeared to present with more dense bony union in regenerating areas, compared to all other treatment and control groups (Fig. 4). Hematoxylin and eosin (H&E) staining revealed a dark red homogeneous and organized formation in the bone defect of the 1B1V modRNA transfected group and the appearance of more osteoblast like cells in the underlying matrix (Fig. 4e, j). In contrast, histological analysis from the control groups, those either treated with empty collagen or receiving no treatment, presented with negligible neobone formation and instead stained pink from fibrous connective tissue with less osteoblast like cells in the indicated areas (Fig. 4a, f, b, g). In single treatment groups, part of the bone defect area was occupied by dark red homogeneous stained bone tissue and part of the bone defect area was occupied by pink dyed fibrous connective tissue indicating limited and incomplete repair (Fig. 4c, h, d, i). Similarly, Masson’s stainings (which stains collagen fibers blue), of the 1B1V modRNA treatment group displayed a unified deep blue staining indicating an advanced phase of bone tissue formation in the defect area (Fig. 4o, t). In the collagen only group and the untransfected scaffold groups the defect was covered with light blue fibrous connective tissue (Fig. 4k, p; l, q, respectively); and the single treatment groups again gave rise to less advanced bone repair (Fig. 4m, r; n, s).

**Fig. 5:**
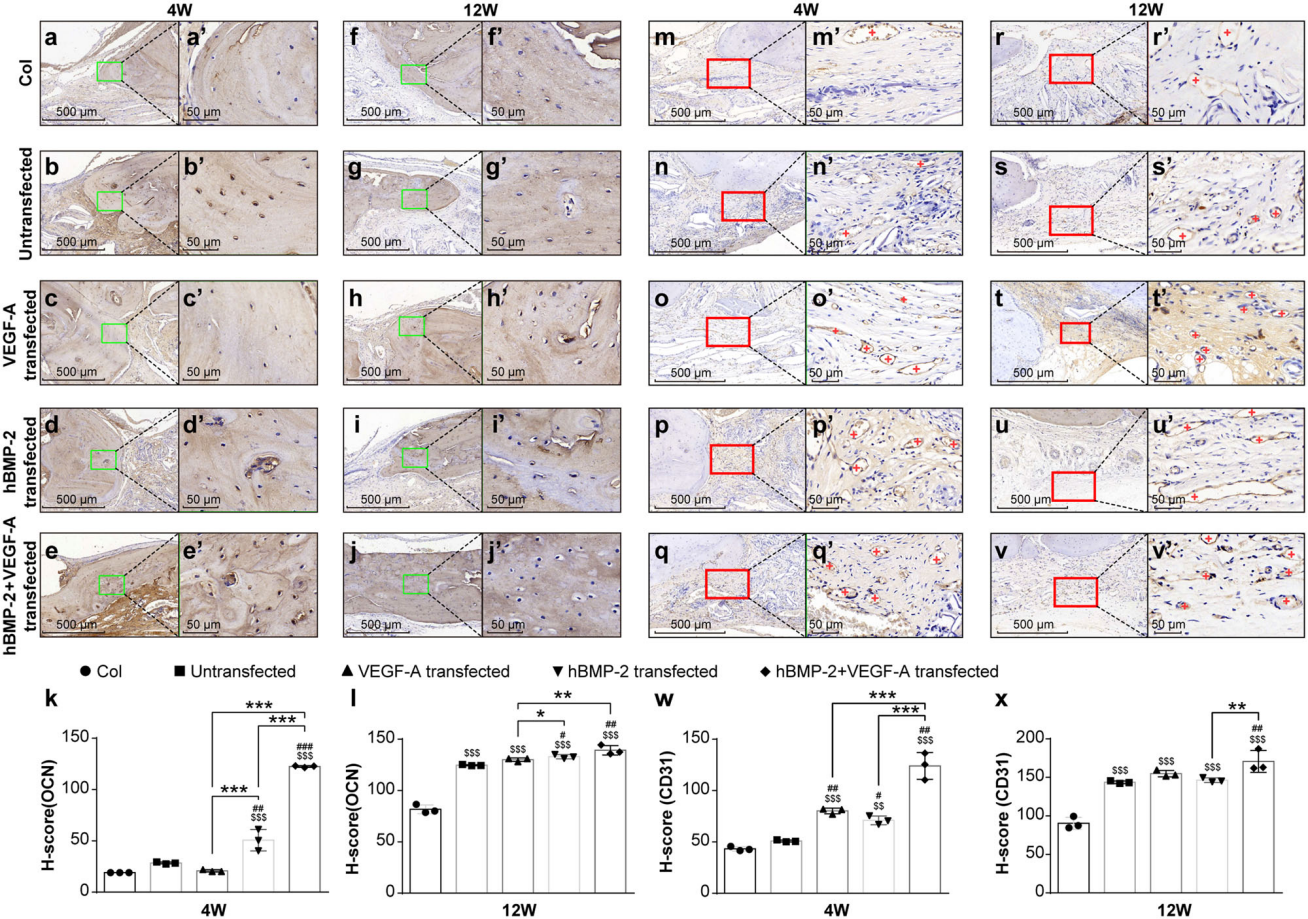
BMSC-scaffolds containing modBMP2 and modVEGF-A intensify the stimulation of new bone formation. **a**–**j** Representative photomicrographs of sectioned and Osteocalcin (OCN) stained bone tissue in the defect areas at 4 weeks (**a**–**e**) and 12 weeks (**f**–**j**) post-treatment, respectively. The rectangular areas in images **a**–**j** correspond to magnified areas in images (**a**’–**j**’). Note the higher quantity of OCN-positive cells in the modBMP2 + modVEGF-A treatment group. **k**, **l** Quantified assessment of OCN-positive cells at the site of regenerating bone in indicated groups at 4 weeks and 12 weeks respectively. **m**–**v** Representative photomicrographs of sectioned and CD31 stained vessels in the defect areas at 4 weeks (**m**–**q**) and 12 weeks (**r**–**v**) post-treatment, respectively. The rectangular areas in images m-v correspond to magnified areas in images (**m**’–**v**’). Note (+) indicates blood vessels in the regenerating area, increased vessel densities in 1B1V treatment group are seen. **w**, **x** Quantification of CD-31 immuno-labeling of the indicated treatment groups at 4 and 12 wks post-injury, respectively. Differences between these groups were calculated by one-way ANOVA test, *n* = 6. Data was represented as the mean ± SD.

To further characterize how the modRNA-transfected BMSCs stimulated neobone formation, we took an immunohistochemical (IHC) analysis approach. First, tissue sections were stained for osteocalcin (OCN) at 4 and 12 wks post-surgery and treatment (Fig. 5a–j). Both the non-transfected control group, as well as the acellular treatment group failed to provide positive stainings for OCN at 4 wks, with remote negligible expression of OCN at 12 wks post-surgery (Fig. 5a, b; f, g, respectively). Interestingly OCN stainings were more abundant in the modBMP2 and 1B1V modRNA groups as early as 4 wks post treatment, indicating efficient and effective osteogenic stimulation from these two treatment regimens over controls (Fig. 5d, e). At 12 wks post treatment, positive stainings for OCN could be seen in all the groups, however OCN activity increased most in the 1B1V modRNA group (Fig. 5 j, j’) suggesting these compounds together better stimulate neo-osteogenic activity than single treatment groups. Quantitative analysis further supported these findings in particular after 4 weeks (Fig. 5k, l).

Next, histological evaluation of CD31 revealed weak and inconsistent signals in the control and acellular treatment groups, indicating poor neovascularization following these treatments (Fig. 5m, n, r, s). All of our treatment groups exhibited positive stainings of CD31 at 4wks and 12 wks post-transplantation, with varying intensities (Fig. 5o–q, t–v). When quantified, higher abundances of CD31^+^ blood vessels were found in the 1B1V modRNA group (*p* < 0.05, Fig. 5w, x). These findings suggest that the combined treatment of BMP-2 and VEGF-A act in concert to stimulate angiogenesis thus improving calvarial bone formation potentially through neovascularization mechanisms.

### BMP-2 and VEGF-A modRNA enhance the molecular profile of osteogenesis

**Fig. 6:**
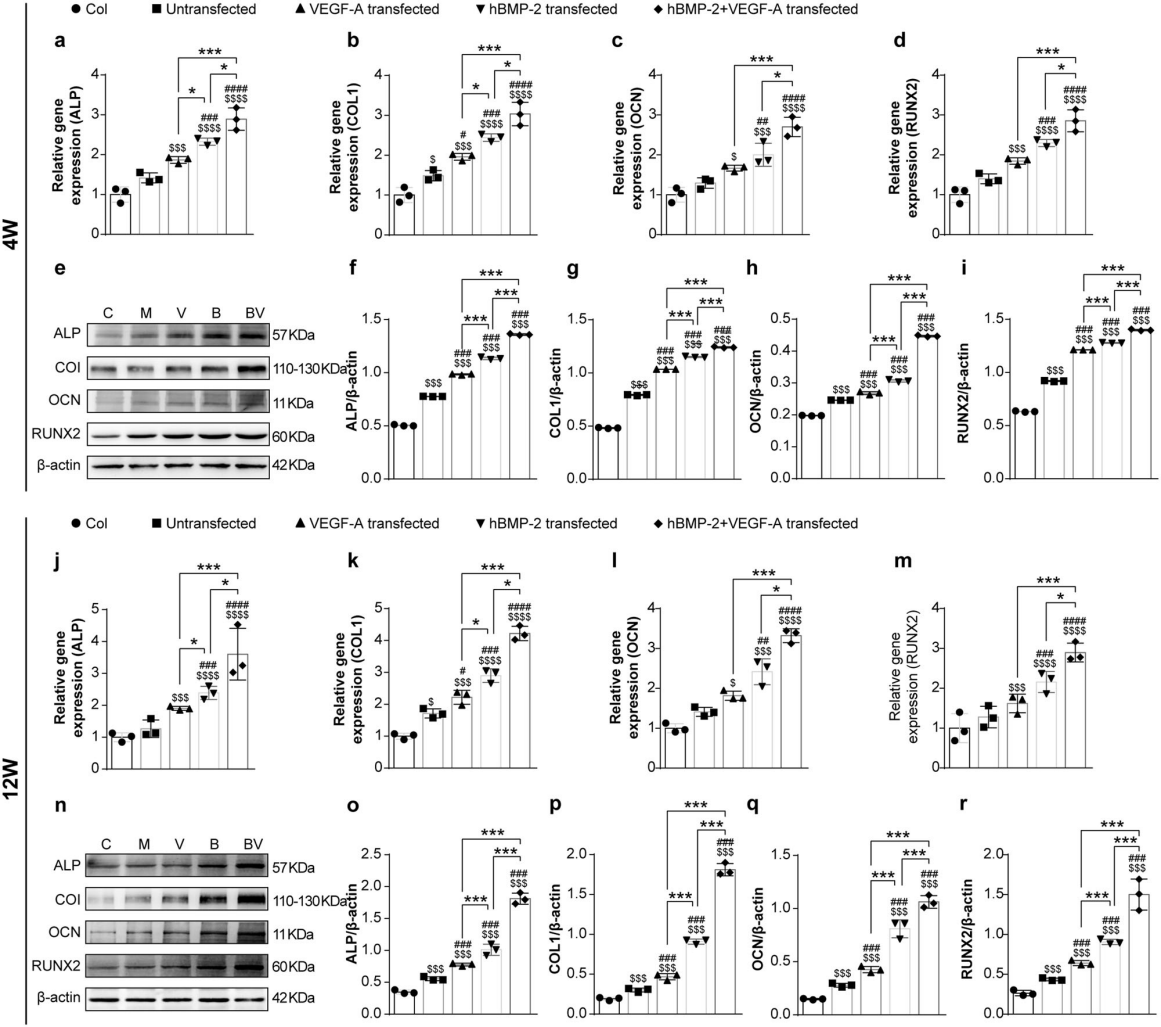
Transcriptional and translational expression patterns of osteogenic genes in regenerating bone ex vivo. Osteogenic related gene expression of ALP, COL1, OCN, and RunX2 were analyzed using qRT-PCR at 4 wks (**a**–**d**) and 12 wks (**j**–**m**) post-implantation. Protein expression of ALP, COL1, OCN, and RunX2 were analyzed using Western blot analysis in untreated and treated groups at 4 wks (**e**–**i**) and 12 wks (**n**–**r**) post-treatment. Dollar ($) indicate difference between acellular collagen scaffold group and treatment groups. Hash (#) indicate difference between non-transfection and transfection groups. Asterisk (*) indicate difference among different modRNA transfection groups. Results for qRT-PCR are normalized to the housekeeping gene (rat GAPDH) and to the empty group, and for western blot, the housekeeping protein is rat β-actin. Values are shown as mean ± SD, ^$/#/^*p < 0.05, ^$$/##/^**p < 0.01, ^$$$/###/^***p < 0.001. *N* = 6, One-way ANOVA followed by Tukey’s multiple comparison.

To investigate the potential of our modRNA-BMSC materials to act on genes that regulate or promote bone formation, we collected RNA and protein from regenerating bone tissue in all our treatment groups (Fig. 6). All the modRNA treatment groups consistently stimulated heightened gene expression patterns of osteogenic genes at 4 and 12 wks post-surgery (Fig. 6a–d; j–m), compared to the acellular group and non-transfection group (*p* < 0.05, *n* = 3). Specifically, the mRNA levels of *ALP*, *COL1*, *OCN*, and *RUNX2* in newly formed bone were increased by the 1B1V treatment group, compared with the control and single administration groups at 4 and 12 wks post-implantation. Similarly, WB results showed that the protein levels of ALP, COL1, OCN, and RUNX2 in newly regenerating bone tissue were higher in the 1B1V treatment group when compared to control groups and single administration groups (Fig. 6e–i, n–r and Supplementary Fig. 11). The increased expression time course of these marker genes was very similar to the time course of osteoblast recruitment and bone formation observed in Figs. 3 and 5. Consequently, BMP2 and VEGF-A modRNA together stimulate endogenous bone repair/growth mechanisms by mediating the osteogenic markers ALP, COL1, OCN, and RUNX2, accelerating bone tissue regeneration synergistically.

### modBMP and modVEGF in vivo correlation studies

Finally, in conjunction with the above studies and in order to investigate how different ratios of the hBMP2 and VEGFA modRNAs could impact functional bone repair, alternative proportions were tested in vivo over a 4wk period (Supplementary Fig. 5). Quantitative analysis of μCT scans revealed only the 3B1V group performed well providing similar levels of neobone formation as was previously seen by the 1B1V group at 4 wks post implantation, (Supplementary Fig. 5a, b and Fig. 3), where the BV/TV was 3.58-fold higher over the untreated group. Histological evaluation of the different groups revealed that the 3B1V group outperformed all other groups based on a bonier union and the appearance of more osteoblast like cells in their underlying matrix (Supplementary Fig. 6a–d). Similarly, the 3B1V group appeared to give rise to more densely contained collagen fibers, an indication of more complete bone growth, made evident by the dark blue homogenous Masson’s stainings (Supplementary Fig. 6e–h).

Next, in order to analyze osteogenic stimulation, tissue sections were stained with OCN. Increased levels of OCN were verified in the 3B1V treatment group over controls and higher than the 1B3V ratio (Supplementary Fig. 7, *P* < 0.05). Furthermore, the analysis of CD31 stainings at 4 wks revealed beneficial induction of neovascularization in the 1B3V group over alternative treatment groups and controls (Supplementary Fig. 7).

Finally, we employed transcriptional and translational profiling as done in Fig. 6, to showcase the effects of different modRNA ratios on regulating neobone formation. Interestingly, when assessing gene expression profiles of *ALP*, *COL1*, *OCN,* and *RUNX2* in newly forming bone tissue at the mRNA level, the 3B1V treatment group gave statistical increases over the 1B3V treatment group and controls (Fig. S8a–d). Likewise, when assessing gene expression at the protein level of these osteogenic marker genes, we again observed increases in the 3B1V treatment group over the 1B3V group, as well as the modLuc and rhBV protein groups (Supplementary Fig. 8e–i and Supplementary Fig. 13). These data provide some indication that manipulating the ratios of BMP and VEGF modRNAs can augment the degree of physiological tissue regeneration.

## Discussion

Adult and fetal BMSCs have previously been applied in experimental research and clinical applications associated with bone tissue engineering^35,36^. Many pre-clinical and clinical studies have highlighted the role of BMSCs with or without biomaterials for clinical bone fracture repair^37,38^. During the past several years more than 3000 patients have received culture-expanded autologous BMSC implantation as a therapy, with no serious adverse effects reported^39,40^. Progress has been made with BMSCs, yet enhancing the clinical applicability requires more consideration. In particular, mechanisms capable of exemplifying targeted efficacy and prolonged benefits while circumventing negative side effects is needed in order to treat bone defects and facilitate bone tissue regeneration.

At the same time, growth factor therapies for bone repair are widely recognized but are accompanied with technological issues around efficacy and safety^41,42^. Often recombinant protein bioactivity is too short to acquire successful regeneration of a large bone defect. BMP2 has previously been a well sought after factor for bone repair as it has been shown to be a powerful growth factor for the induction of osteogenesis^27,43^. However, BMP2 protein or gene therapy studies have been met with several shortcomings, including the relative short half-life of the protein, side effects of ectopic bone formation, inflammation and swelling.

More recently the delivery of BMP growth factors through chemically modified mRNA studies have catalyzed mRNAs unique properties for bone regeneration and repair. Elangovan et al. recently performed elegant work showcasing the benefit of modified BMP mRNA over plasmid DNAs in a rat calvarial defect model. The team employed doses 10fold higher to that of our study (25 μg) and used a polyethlyenimine (PEI)-collagen matrix to deliver the payload. Of interest, they noted good bone repair capacity after just 4 wks, whereas in our study similar repair was seen at 12 wks. The differences here may be attributable to applied doses or the initial overnight inoculation step in our methods where early levels of accumulated protein are lost in the culture prior to transplantation (Fig. 3a). The same team later revealed increased beneficial effects of increased bone matrix deposition using another BMP family member, BMP9. Here, Khorsand et al. applied 50 μg doses of modified BMP mRNA constructs with PEI in an in vivo rat calvarial defect model and showed BMP-9 modRNAs more readily enhanced higher connectivity densities in regenerated bone over BMP2. In another elegant study by Balmayor et al., the team used a femur defect model and demonstrated modified BMP mRNAs within a fibrin gel matrix could induce bone healing in as little as 2 wks when administering “low doses” similar to ours. The uniqueness of our platform over these studies is that we incorporate exogenous BMSCs that not only secrete BMP and VEGF growth factors through modRNA engineering, which can activate neighboring host cell repair mechanisms, but the cells themselves are capable of osteogenic differentiation and may be incorporated into the regenerate. In deed the co-delivery of hBMP-2 and VEGF-A modRNAs were met with remarkable bone healing capabilities. In vitro, the transfection of BMP2 and VEGF-A modRNA led to the extended expression of BMP2 and VEGF-A functional protein in cultivated BMSCs. As a result, BMSCs exhibited increased expression levels of ALP, as well as superior calcium mineralization content identified by alizarin red staining over controls and recombinant protein treatment groups (Fig. 2, Supplementary Fig. 2). In addition, the co-administration of BMP2 and VEGF modRNAs enhanced osteogenic-related gene expression levels more so than recombinant proteins or single-administrated modRNA compounds, demonstrating an advanced molecular mechanism for inducing neobone formation (Fig. 2, Supplementary Fig. 2).

The prospective and beneficial in vitro properties shown by combinatorial modRNA loading of BMSCs were warranted by in vivo animal experiments revealing accelerated healing and enhanced neobone formation within a cranial critical-sized defect in a rat model (Fig. 3, Supplementary Fig. 5). This was made apparent through morpho-histological analyses that revealed osteocalcin and CD31 stainings resembled characteristics more similar to mature bone, over diffuse and discrete stainings seen by controls and single-administration groups (Figs. 4 and 5). Furthermore, we observed the newly regenerated bone tissue resulting from the co-administration groups (i.e., 1B1V and 3B1V) more effectively enhanced expression profiles of genes involved in osteogenic development and repair, a potential indicator of heightened bone quality^44^, over control groups and recombinant proteins. (Fig. 6 and Supplementary Fig. 8).

Importantly, several of the studies mentioned above, as well as our present study rely on LNPs for the successful cellular uptake of exogenous mRNA. Additional studies have indicated issues around cytotoxicity stemming from the use of LNPs^23–25^. Further hindrances of LNP delivery include limitations on cellular uptake based on particle and construct size^45^, as well as endosomal engulfment and/or escape from lysosomal degradation^46^. The use of extracellular vesicles or alternative methods of mRNA delivery such as electroporation should be carefully considered.

Of note, BMP2 and VEGF-A recombinant proteins have been used simultaneously to study their biocompatibility with in vitro model systems^47,48^. Aksel et al. employed a three-dimensional model allowing controlled release of BMP and VEGF growth factors, which enhanced the odontogenic potential of dental pulp stem cells. In addition, Wang et al. showed that BMP2 and VEGF-A proteins possibly act in a synergistic manner, through a p38 MAPK signaling pathway to induce osteogenic maturation in an artificial bone system. Whether VEGF-A and BMP2 modRNAs interact in synergistic fashion by targeting the same pathway or an alternative means of osteogenic cross talk remains to be fully elucidated. Our study emphasizes the therapeutic potential of a combinatorial regenerative system employing modified mRNAs in combination with stem/stromal cells and bone tissue engineering (BTE) biomaterials for advanced bone tissue regeneration in vivo. As previously noted, safety and efficacy is not largely risked when modRNA complexes are delivered to pertinent defective areas together with biomaterial matrix^24,49^. In fact, the combination of a modRNA-biomaterial delivery system may enhance protein secretion and expression over longer periods of time until the support material is completely degraded. We speculate that the co-delivery of multiple growth factors will be a promising aspect in this field and we will focus on these points in our following study. Furthermore, we briefly highlight the importance of fine-tuning the ratios of these growth factors (Fig. 2, Supplementary Fig. 2 and Supplementary Figs. 5–8), however more detailed long-term studies are needed to better address optimal ratios, as well as doses needed to completely restore lost bone tissue.

**Fig. 7:**
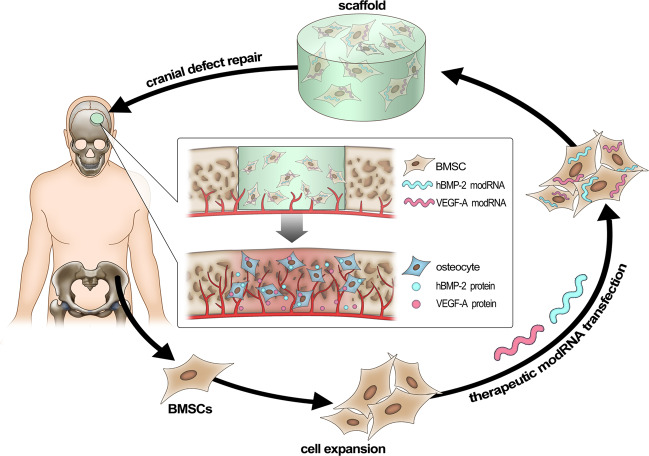
Schematic diagram illustrates the clinical feasibility of combining biomaterials with modRNA-treated BMSCs for bone repair. Our platform favors an autologous cell therapy approach where human BMSCs are isolated from bone marrow of ilium and expanded in vitro. BMSCs are then engineered with modified mRNAs and are inoculated into scaffolds, which are implanted into human bone (cranial) defect sites.

The outlook of this study provides confidence in a therapeutic approach for treating bone defects by taking advantage of chemically modified mRNAs to effectively regenerate bone tissue. Although further exploration is needed to understand the full potential of mRNA doses and combinations to treat bone, we believe these results promote a promising strategy towards bone repair, which may open more doors for utilizing such technologies in future clinical and applied orthopedic research. Furthermore, as shown in Fig. 7, we hypothesize that our system favors an autologous approach to clinical applications of healing bone injuries and fractures. The combination of stem cell therapies with chemically modified mRNAs and biomaterials provides opportunities for developing the next generation of therapeutics for orthopedic medicine.

## Methods

### Cell culture reagents

Dulbecco’s modified eagle medium (DMEM) and fetal bovine serum (FBS) were purchased from Gibco (Thermo Fisher Scientific, USA). Trypsin-EDTA (0.25%, 1× solution), Dulbecco’s phosphate buffered saline (DPBS) and antibiotics (100 U mL^−1^ penicillin G and 0.1 mg ml^−1^ streptomycin) were purchased from Gibco® (Invitrogen™, Grand Island, NY). Cell Counting Kit-8 (CCK-8) was obtained from Dojindo Laboratories (Dojindo, Kumamoto, Japan). Alizarin Red S was purchased from Solarbio^®^Life Sciences, Alkaline phosphatase (ALP) staining kit (BCIP/NBT Alkaline Phosphatase Color Development Kit) was purchased from Beyotime Institute of Biotechnology. Collagen was obtained from Sichuan Mingrang Tech., China and collagen cross-linking agent N-(3-(dimethylamino)-propyl)-N-Ethylcarbodiimide hydrochloride crystalline (EDC) and N-Hydroxysulfosuccinimide sodium salt (NHS) were purchased from Aladdin Industrial Corporation, Shanghai, China. Opti-MEM medium and Lipofectamine^®^ MessengerMAX^TM^ Reagent were obtained from Gibco^TM^ (Invitrogen, CA, USA). Human BMP-2 ELISA kit and human VEGF ELISA kit were obtained from R&D (Quantikine, R&D Systems, USA). D-Luciferin, Sodium Salt was purchased from Yeasen (Shanghai, China). All materials were utilized as received, without any further purification.

### Collagen constructs fabrication and functionalization

Collagen sponges were soaked to become porous as previously described^50^. Briefly, sponges were partially dissolved in 0.1 mol L^−1^ acetic acid and frozen at −80 °C, then freeze-dried at −50 °C under a vacuum for 24 h, subsequently cross-linked by 25 mM EDC and 25 mM NHS with 95% ethanol solution for 24 h, thoroughly washed with 5 wt. % glycine solution and distilled water three times and freeze-dried a second time at −50 °C to obtain collagen scaffold.

### Generation of chemically modified mRNA (modRNA) encoding GFP, mCherry, luciferase, human BMP2 (hBMP-2) and VEGF-A complexes

mRNA was synthesized in vitro using T7 RNA polymerase-mediated transcription from a linearized DNA template, which incorporates the 5’and 3’UTRs and a poly-A tail as previously described^51^. RNA was purified using Ambion MEGA clear spin columns and then treated with Antarctic Phosphatase (New England Biolabs) for 30 min at 37 °C to remove residual 5′-phosphates. Treated RNA was re-purified and quantified by Nanodrop (Thermo Scientific). After purification, modRNA was resuspended in 10 mM Tris HCl, 1 mM EDTA at 1 μg μl^−1^ for use. In the IVT step, uridine was fully replaced by N1-methylpseudouridine. GFP, mCherry, firefly luciferase, and human VEGF-A (165) ORF sequences were the same as previously described^20^. Open reading frame sequence for human BMP-2 modRNA is provided in Supplementary Table 1.

### Isolation and culture of rat BMSCs

All animal experiments were approved by the affiliated Ninth People’s Hospital Institutional Animal Care and Use Committee, Shanghai Jiao Tong University, School of Medicine. Cell isolation process was performed as previously described^32,52^. Marker gene expression profiles of BMSCs were verified using FACS analysis (Fig. S15). Primary BMSCs were harvested from the limbs (ulnae, radii, femora and tibiae) of 1-week old male Sprague-Dawley rats (Shanghai SLAC Experimental Animal Center, Shanghai, People’s Republic of China) and the bone marrow was flushed by 26 G needle attached to a 1 ml disposable aseptic syringe containing DMEM medium. The cells were filtered through a 70-μm filter mesh to remove bone spicules and cell clumps, resuspended in complete low glucose DMEM medium containing 10% FBS, Gibco, 100 IU ml^−1^ penicillin and 100 IU ml^−1^ streptomycin, incubated at 37 °C with 5% CO_2_ in a humidified chamber and were passaged 2–3 times for down-stream experimentation. Characterization of BMSCs that were isolated following the described protocols above were carried out by flow cytometry and multilineage differentiation assays.

### ModRNA preparation and BMSCs transfection

BMSCs were transfected using Lipofectamine® MessengerMAX^TM^ Reagent (Invitrogen, Life Technologies, USA) according to the manufacturer’s instructions. Briefly, BMSCs were seeded at 20 × 10^4^ cells per well in 6-well plates. After incubation of 24 h, cell culture medium was replaced with fresh reduced-serum medium Opti-MEM (Gibco, Life Technologies, USA). ModRNA complexes were formed by using 2.5 µl Lipofectamine® MessengerMAX™ Reagent per 1 µg modRNA. Calculations were performed in order to transfect BMSCs at a final dose of 1 × 10^−5^ µg cell^−1^ modRNA (i.e., 10 pg cell^−1^ for Luciferase, GFP, mCherry, hBMP-2 or VEGF-A). Briefly, in tube A, add 2 µl of modRNA (1 µg µl^−1^) to every 48 µl Opti-MEM and let it sit for 5 min; in tube B, add 5 µl Lipofectamine to every 45 µl Opti-MEM and let it sit for 5 min. Subsequently, mix tube A and B and incubate at room temperature for 15 min. Next, remove old medium from the cells and add 1.5 ml fresh Opti-MEM to each well^53^. Finally, add 100 µl of mixture to each well containing 2 µg modRNA per well. Four hours after transfection, medium was replaced with complete DMEM. The cells were further cultured under standard conditions for up to 7 days until analysis.

### Efficiency and cytotoxicity detection of modRNA complexes

Three different mRNA molecules encoding GFP, hBMP-2 and VEGF-A were modified by the full replacement of uridine-5’-triphosphate with N1-methylpseudo-uridine-5’-triphosphate (m1ψ). GFP modRNA was used as a reporter to demonstrate the transfection efficiency and protein viability of translated transcripts in BMSCs. Twenty-four hours after modGFP transfection the cells were digested with trypsin alongside naïve cells (for control group), centrifuged and resuspended with PBS for the flow cytometry analysis. In addition, cells were fixed with 4% paraformaldehyde for 15 min and the nucleus was stained with Diamidine phenylindole (DAPI), then captured by fluorescence microscope for imaging. Cell proliferation and viability was carried out by transfecting BMSCs with GFP modRNA complexes followed by analysis at 4, 24, 48, and 72 h after transfection using a standard CCK-8 assay performed in triplicates according to the manufacturer’s instructions. Briefly, BMSCs were seeded at 2 × 10^4^ cells per well in 24-well plate, left to recover for 24 h then transfected by GFP modRNA as previously described. Subsequently, the cell monolayer was treated with 200 μl per well of CCK-8 reagent solution (10:1 ratio in serum-free DMEM without phenol red) at different time points post-transfection and then incubated for 2 h under light-protected and standard culture conditions. One hundred microliter medium from each well were transferred into a 96-well plate and the absorbance was determined spectrophotometrically at 450 nm using a microplate reader (SpectraMax® i3x, Molecular Devices, Sunnyvale, CA, USA). Untreated cells were used as a negative control.

### Verification of translation from co-transfected modified mRNAs in BMSCs

To confirm protein expression from two modified mRNA transcripts in a single transfection reaction in BMSCs, we employed a GFP modRNA and an mCherry modRNA construct. BMSCs were divided into 1 of 3 treatment groups: (1) GFP modRNA transfection only (2) mCherry modRNA transfection only (3) GFP modRNA combined with mCherry modRNA transfection. Twenty-four hours post-transfection, cells were analyzed using fluorescence microscopy and flow analysis.

### Expression and quantification of VEGF-A and hBMP-2 using western blotting

BMSCs were seeded at 2 × 10^5^ cells per well in 6-well plates and left to recover for 24 h. Next cells were transfected with VEGF-A modRNA and BMP2 modRNA as previously described.

For western blot analysis, BMSCs were washed with PBS and lysed by protease inhibitor-containing RIPA buffer at 4 °C for 30 min. The lysates were centrifuged and samples were separated by sodium dodecyl sulfate-polyacrylamide gel electrophoresis (SDS‐PAGE) and transferred onto a polyvinylidene difluoride (PVDF) membrane (Millipore, MA). The membrane was blocked with 5% bovine serum albumin (BSA) and probed with the primary antibodies from Abcam. The rat β-actin was used as internal control. The primary antibodies were rabbit anti-VEGF (ab46154) and rabbit anti-BMP-2 (ab214821). After rinsing, the membranes were incubated with horseradish peroxidase-conjugated secondary goat anti-rabbit IgG (1:1000, Beyotime) for 1 h at room temperature. Then the target bands were acquired with an ECL Plus Western Blotting Detection System (GE Healthcare, IL, USA).

### Intracellular expression of VEGF-A and hBMP-2 mRNA quantified by real-time PCR (qRT-PCR)

BMSCs were seeded at 2 × 10^5^ cells per well in 6-well plates and left to recover for 24 h. Next cells were transfected with VEGF-A modRNA and hBMP-2 modRNA as previously described. 24 h post transfection (hpt), total RNA was extracted from BMSCs using EZ-press RNA Purification Kit (EZBioscience, MN, USA), according to the manufacturer’s instructions. Briefly, cells were washed twice with PBS and subsequently lysed by lysis buffer and total RNA was isolated. RNA concentration and purity were determined spectrophotometrically using a microplate reader (SpectraMax® i3x, Molecular Devices, Sunnyvale,CA, USA). cDNA was reverse-transcribed from total RNA (1.5 μg of RNA) by the use of First Strand cDNA Synthesis Kit (EZBioscience, MN, USA) according to the manufacturer’s instructions. The mRNA levels of VEGF-A and hBMP-2 were determined by quantitative real-time PCR (qRT-PCR) analysis with a real-time thermal cycler (Stratagene Mx3000PTM QPCR System, CA, USA) using 2× SYBR Green PCR master mix (EZBioscience, MN, USA). Primers specific for VEGF and BMP-2 genes (Supplementary Table 2) were used.

### Quantification of BMP2 and VEGF-A secretion by ELISA

To quantify the BMP2 and VEGF-A expression following BMSC transfection, the supernatant was sampled at different days post-transfection (dpt) for ELISA analysis using the human BMP-2 ELISA kit (Quantikine, R&D Systems, USA) and human VEGF ELISA kit (Quantikine, R&D Systems, USA), according to the manufacturer’s instructions. The absorbance was measured at 450 nm. Wavelength correction was set at 570 nm. Experiments were performed in triplicates, and the protein content was determined using a standard curve (range: 0-1000 pg ml^−1^ BMP-2 and 0-1000 pg ml^−1^ VEGF).

### ELISA analysis reveals time course of functional protein secretion from modRNA transfected BMSCs

Cell culture supernatants were collected at indicated time points following the transfection of modBMP-2 and modVEGF-A. Serum protein levels were quantified by ELISA (R&D Systems, MN, USA) following the manufacturer’s instructions.

### Endothelial tube formation assay

15–20 ×10^4^ human vascular endothelial cells (HUVEC) were cultured in a 96-well plate on 50 µl of matrigel (BD) together with 100 µl of culture medium taken from either untransfected or VEGF modRNA transfected BMSCs. Three dimensional tubes were visualized and photographed at 4 h and 6 h post-treatment. All experiments were conducted independently, with three replicates in each group.

### In vivo bioluminescence imaging

To evaluate in vivo protein expression kinetics following transplantation of the mRNA-engineered BMSCs, bioluminescence imaging was performed using a Xenogen IVIS Lumina XRMS Series II live animal biophotonic imaging system (Caliper Life Sciences, USA). BMSCs transfected with modRNA encoding luciferase (Luc) were loaded in collagen scaffolds (*n* = 3) and implanted in the defect site. IVIS images were recorded at 1, 2, 3, and 4 days following surgery (*n* = 3 per time point). D-Luciferin, Sodium Salt (CAS 103404-75-7; Yeasen, Shanghai, China) was injected intraperitoneally using a 27 gauge needle at a dosage of 150 mg/kg body-weight under general anesthesia. After 10 min, imaging was performed with an acquiring time of 60 s, exposure time of 0.2 s, binning of 8 and field of view 24. Signals were collected from a defined region of interest (ROI) using the contour ROI tool and measured values are given in Radiance (photons/sec/cm^2^ /steradian). Image analysis was done using the software Living Image (Caliper, USA). At 24 h time intervals following implantation, animals were sedated and non-invasively imaged. Animals with a cranial defect site and a collagen scaffold served as baseline for Luc expression. Signal intensities reported by the modRNA groups were acquired by subtracting the baseline expression.

### Osteogenic differentiation in vitro

In order to enhance osteogenic differentiation, BMSCs were cultured with osteogenic medium following modRNA transfection^32^. The osteogenic differentiation medium (i.e., 10% FBS, 10 mM β-glycerophosphate, 0.05 mM ascorbic acid, and 0.10 μM dexamethasone) was prepared in advance. hBMP-2, VEGF-A, and hBMP-2+VEGF-A modRNA was transfected into the BMSCs as previously described. The medium was exchanged with osteogenic medium 4 h post-transfection for all groups. Transfected cells were sustained under osteogenic conditions for up to 14 days and the medium was changed every 2–3 days. Non-transfected cells served as a control group and were cultured under the same terms and conditions.

### Alkaline phosphatase activity assay and staining

Alkaline phosphatase activity (*n* = 3) was assessed utilizing an ALP assay kit according to the manufacturer’s protocol, as described previously^54,55^. After osteogenic induction culture for 7 days, the cell layers were washed gently with cold PBS and digested with 0.25% trypsin-EDTA for 1 min. The cell lysates were centrifuged at 1500 rpm for 5 min at 4 °C. Then cell precipitate was lysed in 200 μl of 0.2% Triton X-100 for 30 min. Next, 30 μl of the supernatant was mixed with 150 μl of the working solution following the manufacturer’s protocol (Nanjing Jiancheng Bioengineering Institute, Nanjing, China). The substrate of ALP, namely, the resultant of p-nitrophenol from p-nitrophenylphosphate, was evaluated by measuring the absorbance at 520 nm with a microplate reader (SpectraMax® i3x, Molecular Devices, Sunnyvale, CA, USA). The ALP activity was calculated by the ratio of experimental samples to standard and was expressed by milligrams of p-nitrophenol produced per minute per gram of protein (king unit per gprot). Furthermore, BMSCs were subject to alkaline phosphatase staining at 7 dpoi as described previously^56^. Fixed cells were stained with the staining mixture of BCIP and NBT for 30 min at 37 °C. The staining solution was washed with PBS carefully and cells were photographed and analyzed using an inverted microscope. Areas that were stained in purple were considered as positive.

### Alizarin red staining and quantification

To detect mineralization, BMSCs were subject to Alizarin red staining at 14 dpoi, as described previously^57^. After removing the medium, the cells were washed twice with PBS and fixed with 4% paraformaldehyde for 30 min. Then cells were rinsed with PBS twice, and stained with 0.2% Alizarin Red S solution (pH 4.2) for 5 min, followed by three times PBS wash extensively to remove unspecific staining or verisimilar sediments. Calcified nodules are stained as red spots and were photographed by a microscope. To quantify the calcium deposition, after washing with dH_2_O, the stain was solubilized with 10% cetylpyridinium chloride monohydrate (CPC, Sigma-Aldrich) in 0.1 M PBS (pH 7.0) by shaking for 15 min and the absorbance was read at 570 nm^58^. The calcium deposition is expressed as optical density (OD).

### qRT-PCR and western blotting analysis for osteogenesis in vitro

At 7dpoi total RNA was extracted from transfected BMSCs using EZ-press RNA Purification Kit, according to the manufacturer’s instructions as previously described^59^. 1.5 μg of RNA was reverse transcribed. The osteo-related gene expression levels were determined by qRT-PCR analysis with a real-time thermal cycler using 2× SYBR Green PCR master mix. For qRT-PCR, primers specific for alkaline phosphatase (ALP), type I collagen (COL1), osteocalcin (OCN) and runt-related transcription factor 2 (RUNX2) genes (Supplementary Table 3) were used.

Alternatively, cell samples were harvested at 3dpoi, washed with PBS and lysed by protease inhibitor-containing RIPA buffer at 4 °C for 30 min, followed by western blot. The rat β-actin was used as internal control. The primary antibodies were rabbit anti-ALP (ab95462), rabbit anti-COL1 (ab34710), rabbit anti-OCN (ab93876), and rabbit anti-RUNX2 (ab192256) (all from abcam). The secondary antibody was HRP-conjugated goat anti-rabbit IgG (1:1000, Beyotime). Then the target bands were acquired with an ECL Plus Western Blotting Detection System (GE Healthcare, IL, USA).

### Preparation of rat modRNA-BMSC/scaffold constructs and surgical procedures

The modRNA-transfected cells were trypsinized 4 h post-transfection, centrifuged and resuspended in osteoblast inducing conditional medium. The cells (5 × 10^6^ cells per ml) were evenly seeded into cross linked collagen scaffold in the 48-well plate, 50 μl per scaffold, thus 2.5 × 10^5^ cells per scaffold, (2 μg modRNA in total was transfected into 20 × 10^4^ cells). Osteogenic medium was added after 4 h incubation at 37 °C, and the BMSC per scaffold constructs were incubated overnight prior to implantation^60^.

8-week-old male Spraguee Dawley rats were purchased from the animal experimental center of Shanghai ninth people’s hospital (China), where they were housed and looked after in the experimental animal house. Two weeks later, they were anesthetized by intraperitoneal injection of 10% chloral hydrate (4 mg kg^−1^ body weight). In order to expose the parietal bone adequately, a sagittal incision (1.8 cm) in the scalp was made and the pericranium was removed by gentle scraping. The skull was washed by sterile phosphate buffered solution (PBS). Two critical-sized (5 mm in diameter) defects were created on both sides of the sagittal suture on the parietal bone, without disturbing the underlying dura and superior sagittal sinus vein, using a sterile medical bone drill. The constructs were implanted into the defects and gently pressed. The incision was carefully closed in layers by a 5-0 biodegradable suture and a 6-0 silk thread. Five treatment regimens were employed in this study, where defects were implanted with one of the following: (1) empty collagen scaffold (*n* = 6); (2) naïve BMSC loaded collagen scaffold (*n* = 6); (3) BMSCs treated with modRNA encoding VEGF-A-loaded collagen scaffold (*n* = 6); (4) BMSCs treated with modRNA encoding hBMP-2-loaded collagen scaffold (*n* = 6); and (5) BMSCs treated with modRNA encoding hBMP-2+VEGF-A-loaded collagen scaffold (*n* = 6).

### Optimization of BMSC osteogenic differentiation employing different ratios of hBMP-2 and VEGF-A modRNA in the absence of dexamethasone

Different ratios of hBMP-2 and VEGF-A modRNA co-transfected BMSCs were tested in the absence of dexamethasone to explore osteogenic differentiation potential. In addition, BMSCs were treated with VEGF-A and BMP-2 recombinant human proteins (cat #100-20-2 and cat #120-02-2, respectively, PeproTech) with the same osteogenic differentiation medium (i.e., 10% FBS, 10 mM β-glycerophosphate and 0.05 mM ascorbic acid). Recombinant protein doses were set by adding the equivalent cumulative levels of protein found in modRNA transfections. Alkaline phosphatase activity assay and staining was carried out after osteogenic induction culture for 7 days, as described previously^54^. Alizarin red staining and quantification was carried out to detect mineralization at 14 days post transfection (dpt). Molecular analysis for major osteogenic genes were carried out using qRT-PCR and western blotting at 7dpt.

### In vivo surgical procedures employing different ratios of hBMP-2 and VEGF-A modRNA in the absence of dexamethasone

8-week-old male Spraguee Dawley rats were purchased from the animal experimental center of Shanghai ninth people’s hospital (China), where they were housed and looked after in the experimental animal house. Cranial defects were performed as described in previous methods section. Four treatment regimens were employed in this study, where defects were implanted with one of the following: (1) BMSCs treated with modRNA encoding Luciferase-loaded collagen scaffold (*n* = 3); (2) BMSCs treated with modRNA encoding hBMP-2: VEGF-A (in a 3:1 ratio)-loaded collagen scaffold (*n* = 3); (3) BMSCs treated with modRNA encoding hBMP-2: VEGF-A (in a 1:3 ratio)-loaded collagen scaffold (*n* = 3); and (4) BMSCs treated with recombinant protein BMP-2+VEGF-A (1:1)-loaded collagen scaffold (*n* = 3). At 4 weeks post transplantation, we sacrificed the rats and removed the calvarial bones and four samples per group were harvested. The samples were processed by micro-CT, were histologically and immunohistochemically assessed, and we carried out qRT-PCR and western blot analyses for newly regenerated bone tissue. For immunohistochemical staining, IOD (integrated optical density) and AREA (area of selected region) of each photo were obtained by analyzing with software. IOD can reflect the total protein expression in the selected AREA. AO (average optical density value, AO = IOD/AREA) can be calculated. The higher the AO value, the higher the positive expression level.

### Micro computed tomography (μCT)

We sacrificed the rats and removed the calvarial bones at 4 and 12 weeks post-surgery^61^ and four samples per group were harvested. The samples were fixed in 4% paraformaldehyde for 24 h and then scanned using a SkyScan-1176 micro-computed tomography (μCT) (Bruker micro CT, Belgium) system. Scans were performed using 17.93 μm voxel size, 90KV, 278 μA, and 0.5 degrees rotation step (180 degrees angular range). For bone analysis, micro-CT evaluation was performed on a 5 mm diameter circle, 1 mm height cylinder region in the defect area. The 1.6 version of NR econ software was used for 3D reconstruction and viewing of images. After 3D reconstruction the 1.13 version of CT software was used for bone analysis. The index including directly measured bone volume (BV, mm^3^), tissue volume (TV, mm^3^), and the ratio BV/TV (%), were calculated for the bone formed in the defect area.

### Histological and immunohistochemical staining

After μCT scanning, the removed calvarial bones were decalcified with mild EDTA-decalcifier-solution (Boster Biological Technology Co., Ltd), followed by paraffin embedding and sectioning. The sections (10 μm thick) from mid-defect regions were stained with H&E or subjected to Masson trichrome staining, which detects the collagen formation activity and is a sign of bone remodeling and bone healing. Briefly, the sections were deparaffined and rehydrated for immunohistochemical staining of osteocalcin (OCN) or CD31. The antigen retrieval was performed by incubation with trypsin for 20 min at 37 °C. The primary antibodies were mouse anti-rat OCN (1:150 dilution, abcam) or anti-CD31 MAb (1:200 dilution, Servicebio). The secondary antibody was goat anti-mouse HRP-conjugated MAb (1:1000 dilution, abcam). For Masson, OCN and CD31 staining, the nuclei were stained with Gill Hematoxylin (Sigma) at the end of procedures. Expression of OCN and CD31 were quantified by Image J software, as described previously^62^.

### qRT-PCR and western blot assay for new regenerated bone tissue

Newly formed bone tissues in the defect areas of the crania were sampled after the rats were euthanized 4 and 12 wks after surgery. Total RNA was extracted from the bone tissue using a trizol reagent (Invitrogen, CA) according to the manufacturer’s instructions. The uniform real‐time (RT) primer was used for the reverse transcription and quantitative RT-PCR was performed using a real-time thermal cycler (Stratagene Mx3000PTM QPCR System, CA, USA) and 2× SYBR Green PCR master mix (EZBioscience, MN, USA).

Whole bone tissue isolations were extracted using a total protein lysis buffer. Tissue samples were lysed in RIPA lysis buffer (Sigma-Aldrich) containing a proteasome inhibitor (Beyotime) and then followed by western blot as previously described. Finally, the target bands were acquired with an ECL Plus Western Blotting Detection System (GE Healthcare, IL, USA).

### Statistics and reproducibility

The statistical analyses were performed using GraphPad Prism Version 7.00 (GraphPad Software, CA, USA). Results are expressed as mean ± standard deviation (SD) and unpaired Student’s t-test was employed to analyze the differences between the untransfected and transfected group. One-way ANOVA were performed to analyze comparison of multiple groups. ANOVA analyses were corrected for multiple comparison by Tukey (Gaussian distribution) and Kruskal–Wallis or Dunn’s (non-Gaussian distribution) tests. All data that are representative performed at least 3 independent experiments, and the differences were considered statistically different at the levels of *p < 0.05, **p < 0.01 and ***p < 0.001. *p* < 0.05 represents statistical significance.

### Reporting summary

Further information on research design is available in the Nature Research Reporting Summary linked to this article.

## Supplementary information

Supplementary Information

Description of Additional Supplementary File

Supplementary Data 1

Reporting Summary

## Data Availability

The raw source data for the main figures are included in Supplementary Data 1. Any other data not included in the paper or supplementary materials is available from the authors upon reasonable request. The corresponding author, Dr. Wei Fu, will provide the requested data.
