# Supplementary material for: BMP-2 and VEGF-A modRNAs in collagen scaffold synergistically drive bone repair through osteogenic and angiogenic pathways
Source: Commun Biol. 2021 Jan 19;4:82. doi: 10.1038/s42003-020-01606-9 (PMC7815925; doi:10.1038/s42003-020-01606-9)

Supplementary Tables and Figures

Supplementary Table 1. Open reading frame sequence for construction of human BMP-2 modRNA

Human BMP-2	ATGGTGGCCGGGACCCGCTGTCTTCTAGCGTTGCTGCTTC<br>CCCAGGTCCTCCTGGGCGGCGCGGCTGGCCTCGTTCCGG<br>AGCTGGGCCGCAGGAAGTTCGCGGCGGCGTCGTCGGGCC<br>GCCCCTCATCCCAGCCCTCTGACGAGGTCCTGAGCGAGTT<br>CGAGTTGCGGCTGCTCAGCATGTTCGGCCTGAAACAGAG<br>ACCCACCCCCAGCAGGGACGCCGTGGTGCCCCCCTACAT<br>GCTAGACCTGTATCGCAGGCACTCAGGTCAGCCGGGCTC<br>ACCCGCCCCAGACCACCGGTTGGAGAGGGCAGCCAGCCG<br>AGCCAACACTGTGCGCAGCTTCCACCATGAAGAATCTTT<br>GGAAGAACTACCAGAAACGAGTGGGAAAACAACCCGGA<br>GATTCTTCTTTAATTTAAGTTCTATCCCCACGGAGGAGTT<br>TATCACCTCAGCAGAGCTTCAGGTTTTCCGAGAACAGAT<br>GCAAGATGCTTTAGGAAACAATAGCAGTTTCCATCACCG<br>AATTAATATTTATGAAATCATAAAACCTGCAACAGCCAA<br>CTCGAAATTCCCCGTGACCAGACTTTTGGACACCAGGTTG<br>GTGAATCAGAATGCAAGCAGGTGGGAAAGTTTTGATGTC<br>ACCCCCGCTGTGATGCGGTGGACTGCACAGGGACACGCC<br>AACCATGGATTCGTGGTGGAAGTGGCCCACTTGGAGGAG<br>AAACAAGGTGTCTCCAAGAGACATGTTAGGATAAGCAGG<br>TCTTTGCACCAAGATGAACACAGCTGGTCACAGATAAGG<br>CCATTGCTAGTAACTTTTGGCCATGATGGAAAAGGGCAT<br>CCTCTCCACAAAAGAGAAAAACGTCAAGCCAAACACAA<br>ACAGCGGAAACGCCTTAAGTCCAGCTGTAAGAGACACCC<br>TTTGTACGTGGACTTCAGTGACGTGGGGTGGAATGACTG<br>GATTGTGGCTCCCCCGGGGTATCACGCCTTTTACTGCCAC<br>GGAGAATGCCCTTTTCCTCTGGCTGATCATCTGAACTCCA<br>CTAATCATGCCATTGTTCAGACGTTGGTCAACTCTGTTAA<br>CTCTAAGATTCCTAAGGCATGCTGTGTCCCGACAGAACTC<br>AGTGCTATCTCGATGCTGTACCTTGACGAGAATGAAAAG<br>GTTGTATTAAAGAACTATCAGGACATGGTTGTGGAGGGT<br>TGTGGGTGTCGCTAG

Supplementary Table 2. Human primers used in qRT-PCR analysis.

Primers	Sequence (5'-3')	Product size(bp)
GAPDH-F	GGGAAGGTGAAGGTCGGAGT	105
GAPDH-R	GGGGTCATTGATGGCAACA	
VEGF-F	TGTGCCCACTGAGGAGTC	102
VEGF-R	CATTTGTTGTGCTGTAGGAAG	
BMP2-F	ACTCGAAATTCCCCGTGACC	144
BMP2-R	CCACTTCCACCACCAATCCA	

Supplementary Table 3. Rat primers used in qRT-PCR analysis.

Primers	Sequence (5'-3')	Product size(bp)
GAPDH-F	GCCATCAACGACCCCTTCAT	136
GAPDH-R	AGATGGTGATGGGTTTCCCG	
ALP-F	CCTTAAGGGCCAGCTACACC	100
ALP-R	AGCGTTGGTGTTGTACGTCT	
COI-F	TGGTGAGACGTGGAAACCTG	193
COI-R	CTTGGGTCCCTCGACTCCTA	
OCN-F	GGTGGTGAATAGACTCCGGC	117
OCN-R	AGCTCGTCACAATTGGGGTT	
RUNX2-F	ACTACTCTGCCGAGCTACGA	86
RUNX2-R	GCTCCGGCCTACAAATCTCA	

Supplementary Fig. 1 Translation and expression kinetics of modRNA in transfected BMSCs

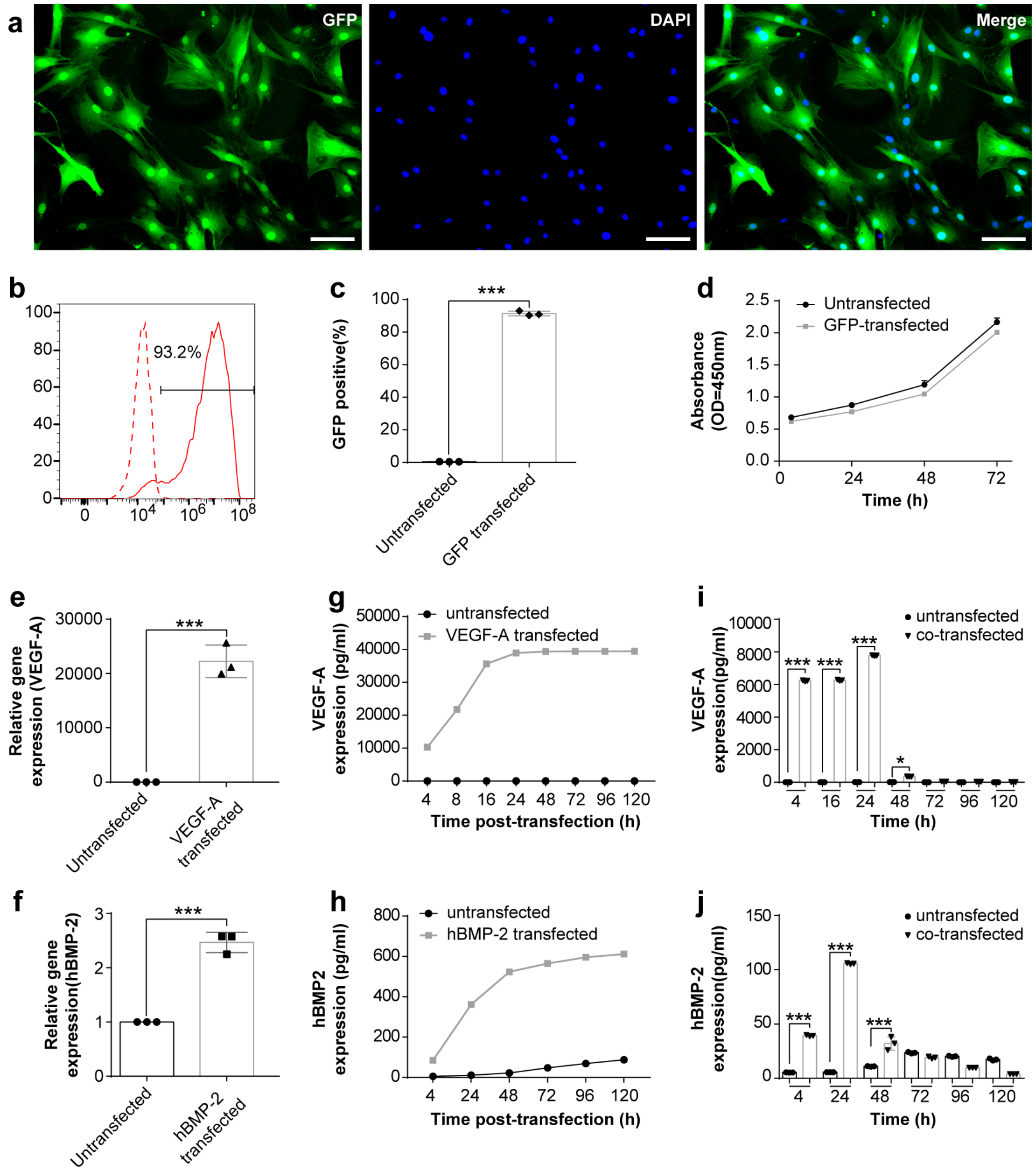

(a) Representative fluorescence microscopy images of BMSCs taken 24hpt with GFP modRNA stained with DAPI. The applied modRNA dose was 10 pg per cell. The scale bars represent 125μm. (b) GFP expression of BMSCs after transfection with GFP modRNA was determined by flow cytometry. (c) Representative flow cytometry histograms for BMSCs 24 hours post-transfection with GFP modRNA. (d) Effect of modRNA transfection on BMSC proliferation. (e, f) qPCR determination of gene expression in BMSCs transfected with VEGF-A and BMP2 modRNA for 24h respectively. (g) and (h) Time course of BMP2 and VEGF-A protein expression and accumulation from BMSCs transfected with modBMP2 and modVEGF-A complexes, respectively. (i) and (j) Time course of BMP2 and VEGF-A protein expression and

accumulation from BMSCs co-transfected with modBMP2 and modVEGF-A. Significant differences between control and transfection are indicated by (*) and (***), respectively.

Supplementary Fig. 2. Cellular and molecular detection of *in vitro* osteogenesis following different ratios of hBMP-2 and VEGF-A co-transfection in BMSCs

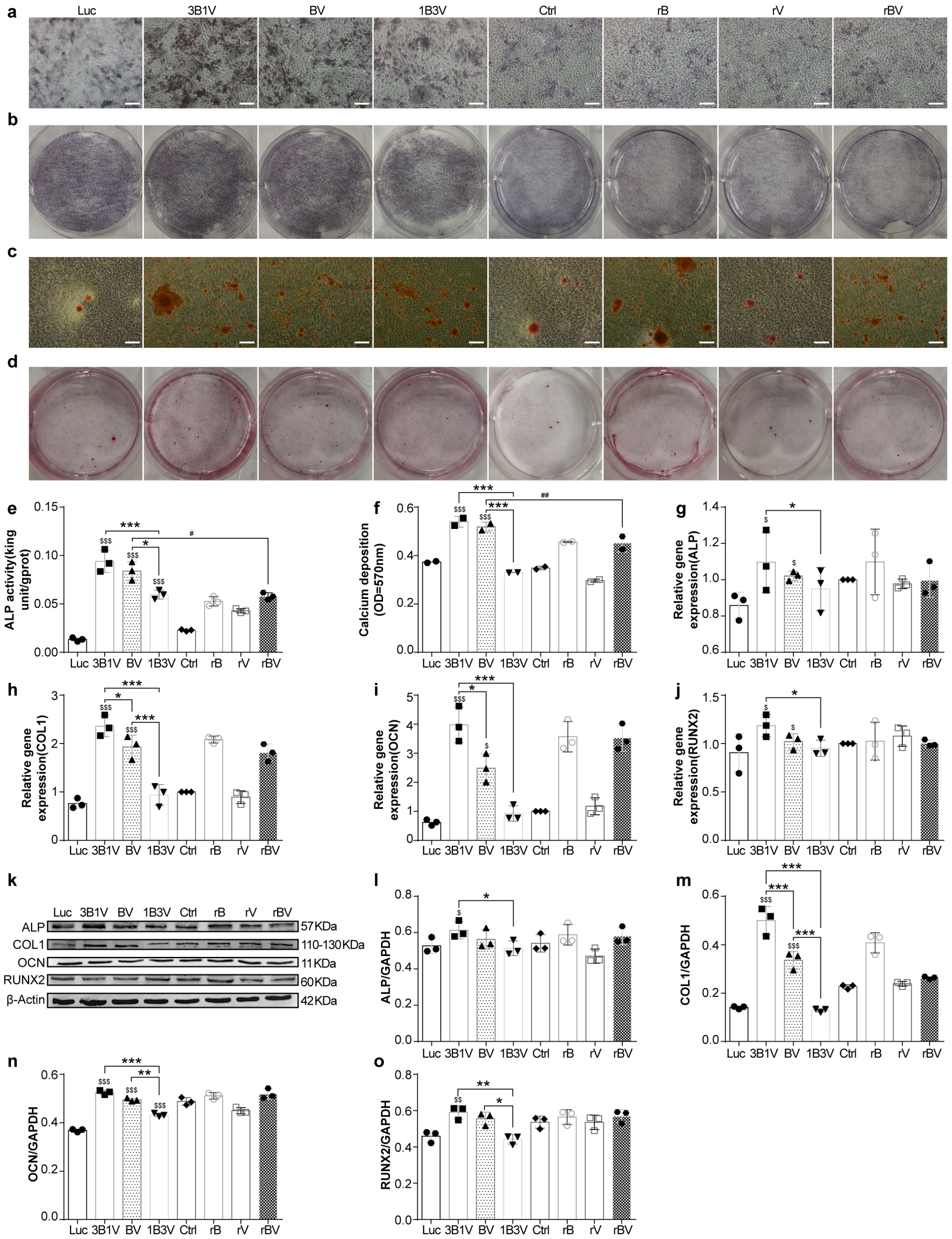

(a) Alkaline phosphatase (ALP) staining of BMSCs at 7 days post-transfection in different ratios of modRNA and recombinant proteins. (b) Gross appearance of ALP staining. Note appearance of ALP expression appears strongest in 3B1V and 1B1V

modRNA groups. (c) Detection of mineralized matrix using alizarin red staining 14 days post-transfection in indicated modRNA and recombinant protein treatment groups. (d) Gross appearance of alizarin red staining 14 days post transfection. The scale bars represent 250µm. Quantification of ALP activity (e) 7dpt and quantification of alizarin red staining (f) 14dpt. (g-j) Fold increase in expression of (g) ALP, (h) Collagen Type I (COL 1), (I) Osteocalcin (OCN), (j) Runt-related transcription factor 2 (RunX2) at 7dpt determined by qRT-PCR analysis. Representative western blot assessment (k) and quantification of protein expression of ALP (l), COL1 (m), OCN (n) and RUNX2 (o) following transfection of indicated modRNA and recombinant proteins treatments. ($) Indicate significant difference between hBMP-2, VEGF-A modRNA transfection and luciferase modRNA transfection groups. ($) Indicate significant difference between modRNA transfection and recombinant proteins treated groups. (*) Indicate significant difference among different ratios of hBMP-2 and VEGF-A modRNA transfection groups. Results for qRT-PCR are normalized to the housekeeping gene (rat GAPDH) and to the control group. Results for western blot are normalized to the housekeeping gene (rat β-actin) and to the untransfected cells. Values are shown as mean ± SD, $^{\$/}$*p<0.05, $^{\$\$/}$**p<0.01, $^{\$\$\$/}$***p<0.001. N=3, One-way ANOVA followed by Tukey's multiple comparison.

Supplementary Fig. 3 Induction of angiogenesis in HUVECs following the addition of modRNA transfected supernatant.

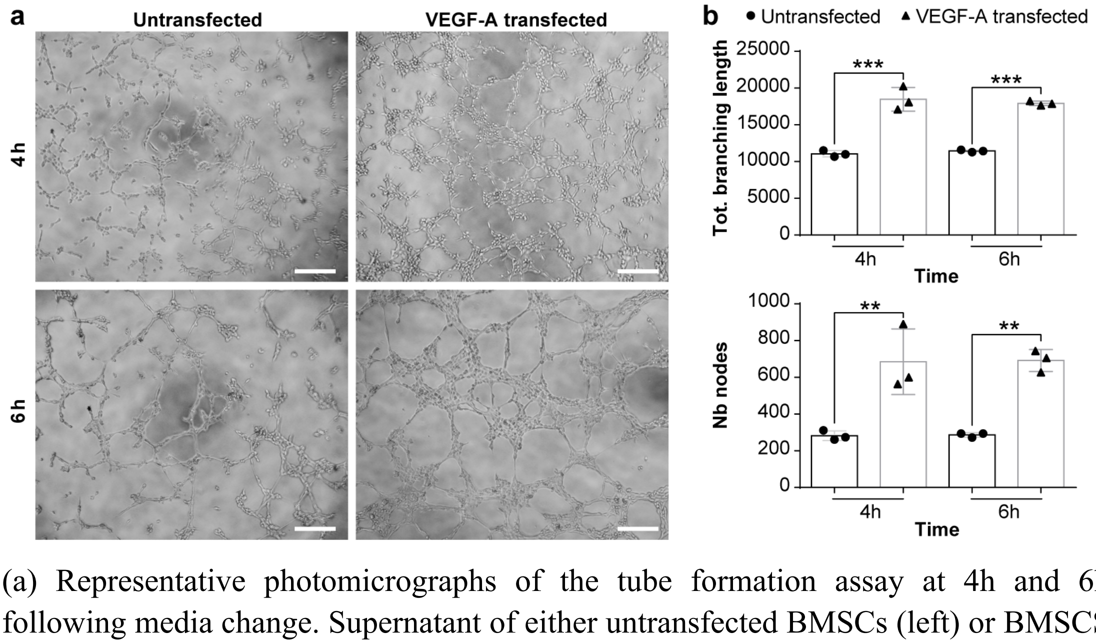

(a) Representative photomicrographs of the tube formation assay at 4h and 6h following media change. Supernatant of either untransfected BMSCs (left) or BMSCS transfected with modVEGF-A (right) was used. (b) Quantitative analysis of the tube formation assay between VEGF-A modRNA transfected and untransfected groups, P<0.05.

Supplementary Fig. 4 *In vivo* expression profile and time course of Luc modRNA-transfected BMSCs in bone

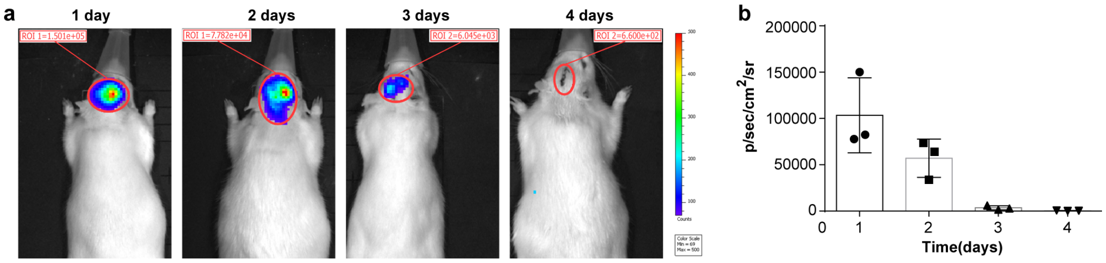

(a) Daily IVIS imaging and recordings demonstrate positive luciferase expression following Luc modRNA-treated BMSC transplantation. (b) Luc levels were measured as photons/sec/cm^2/sr. Positive Luc protein expression could be assayed for three days. At day 4, protein expression dropped to baseline values.

Supplementary Fig. 5. BMSCs engineered with alternative ratios of modRNAs in an *in vivo* rat cranial defect model

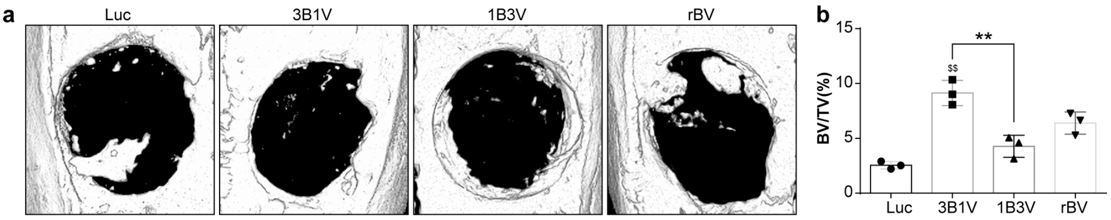

(a) Representative images reconstructed from 3D μCT scans reveals bone tissue regeneration at 4wks after treatment. Note, area of callus formation appears greater in 3B1V treatment group. (b) Quantified bone volume fraction (BV/TV) of regenerated bone at 4wks post-implantation. Significant differences between modRNA treatments and recombinant protein group (rBV) were assessed by one-way ANOVA followed by Tukey's multiple comparison. ($) Indicate significant difference between 3B1V transfection and luciferase modRNA transfection groups. ($) Indicate significant difference between modRNA transfection and recombinant proteins treated groups. (**) Indicate significant difference among different ratios of hBMP-2 and VEGF-A modRNA transfection groups. (Values are expressed as mean ± SD, $^{\$/\#/}$*p<0.05, $^{\$\$/\#\#/}$**p<0.01, $^{\$\$\$/\#\#\#/}$***p<0.001).

Supplementary Fig. 6. Histological evaluation of bone defects and repair following injury and scaffold implantation of ratio test.

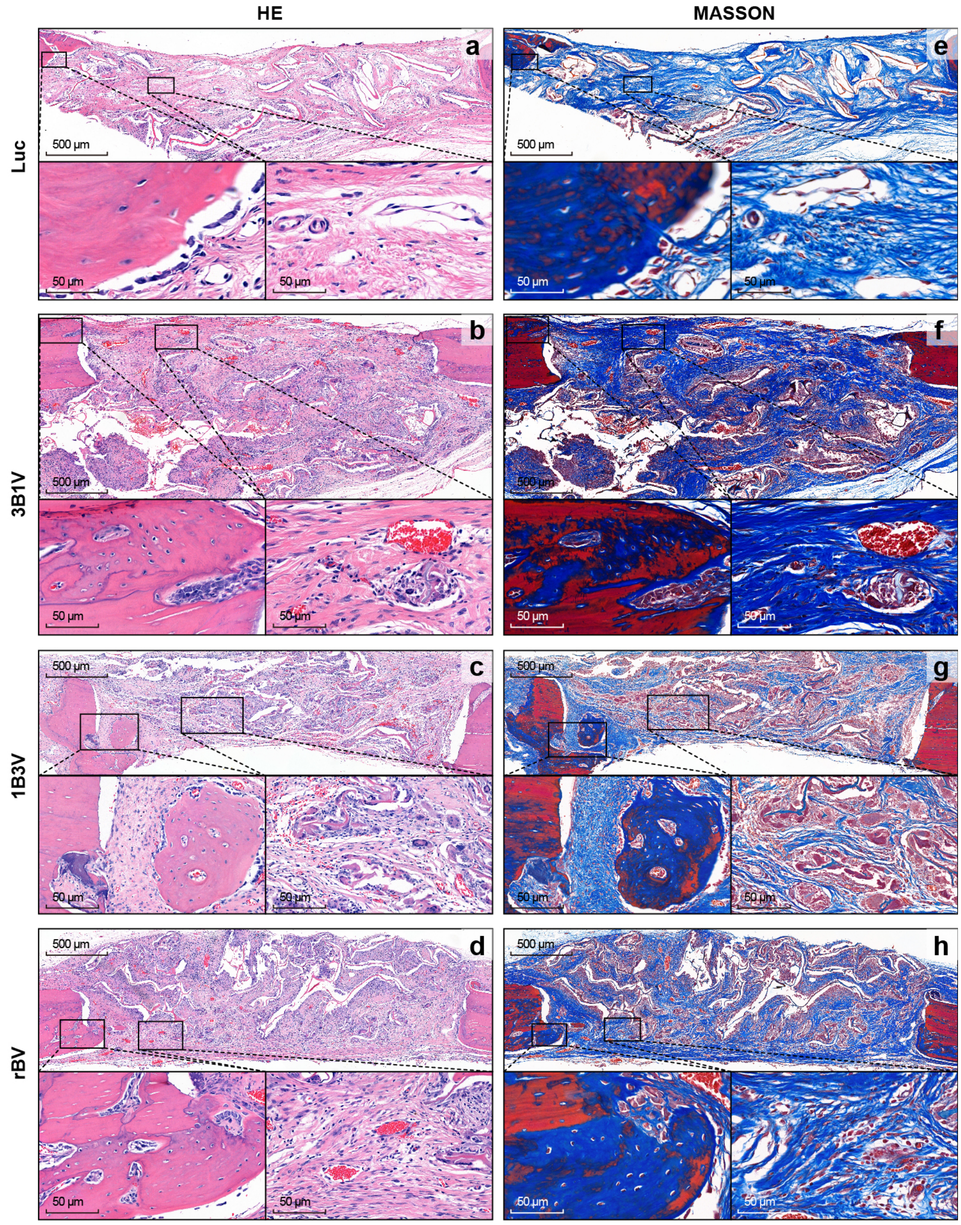

Hematoxylin and eosin (H&E) staining of the cranial defect location at 4wks (a-d) post-implantation. (e-h) Masons trichrome staining of the cranial defect location at 4wks post-treatment. Different increased levels of bone-forming osteoids were detected in modRNA and recombinant proteins treatment groups, as indicated by red staining. The most significant level of bone repair was found in the group of scaffolds embedded with 3B1V modRNA transfected BMSCs compared to other groups.

Supplementary Fig. 7. BMSC-scaffolds containing 3B1V ratio intensify the stimulation of new bone formation.

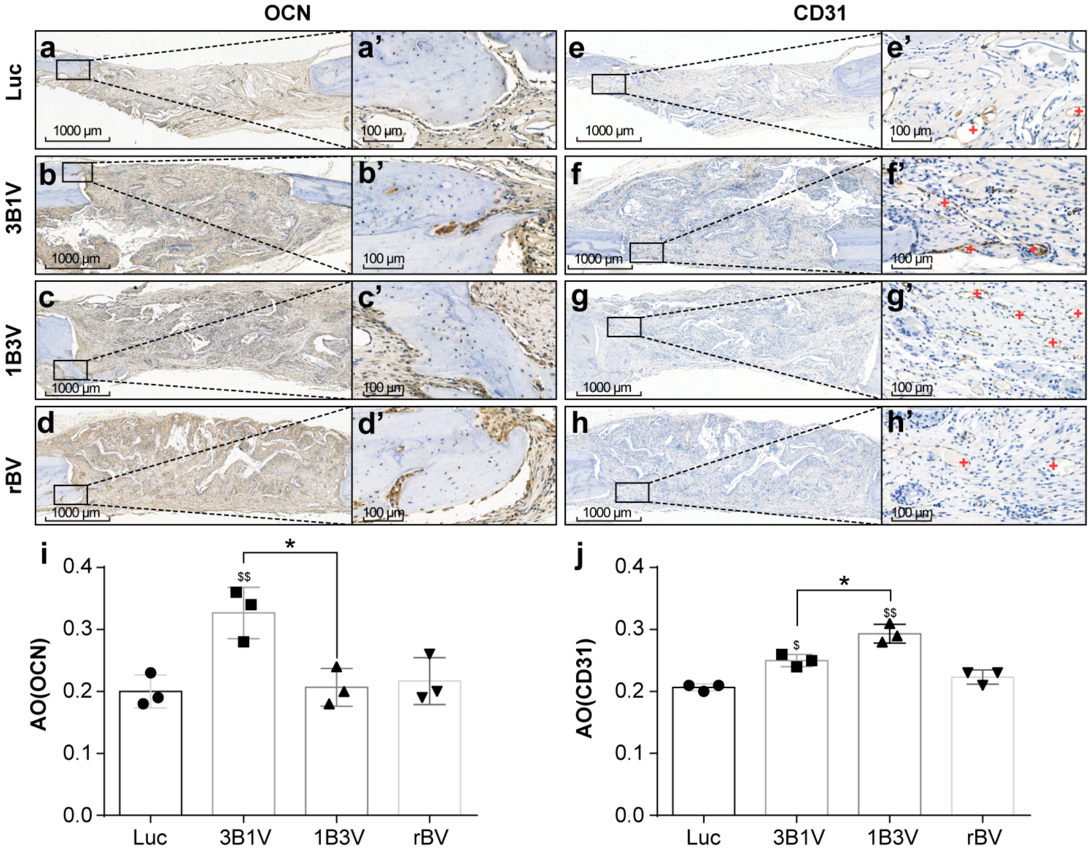

(a-d) Representative photomicrographs of sectioned and Osteocalcin (OCN) stained bone tissue in the defect areas at 4wks post-treatment. The rectangular areas in images a-d correspond to magnified areas in images (a'-d'). Note the higher quantity of OCN-positive cells in the 3:1 treatment group. (i) Quantified assessment of OCN-positive cells at the site of regenerating bone in indicated groups at 4wks. **(**e-h**)** Representative photomicrographs of sectioned and CD31 stained bone sections in the defect areas at 4wks post-treatment. The rectangular areas in images e-h correspond to magnified areas in images (e'-h'). Note increased vessel densities in 1B3V treatment group. (j) Quantification of CD-31 immunolabelling of the indicated treatment groups at 4wks. Differences between these groups were calculated by one-way ANOVA test, n=3. Data was represented as the mean ± SD.

Supplementary Fig. 8. Transcriptional and translational expression patterns of os teogenic genes in regenerating bone tissue.

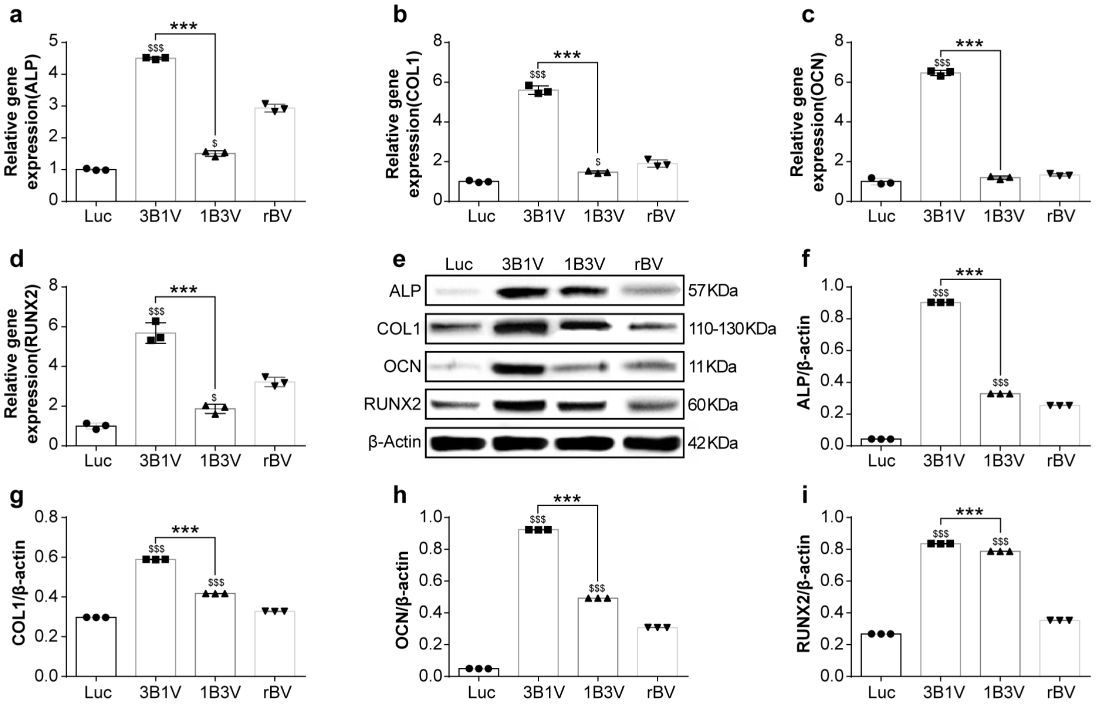

(a-d) Osteogenic related gene expression of ALP, COL1, OCN, and RunX2 were analyzed using qRT-PCR at 4wks post-implantation. (e-i) Detection of protein levels for ALP, COL1, OCN, and RunX2 in regenerating bone tissue using Western blot analysis at 4wks post-treatment. ($) Indicate significant difference between hBMP-2, VEGF-A modRNA transfection and luciferase modRNA transfection groups. (*) Indicate significant difference among different ratios of hBMP-2 and VEGF-A modRNA transfection groups. Results for qRT-PCR are normalized to the housekeeping gene (rat GAPDH) and to luciferase modRNA transfection group, and for western blot, the housekeeping protein is rat β-actin. Values are shown as mean ± SD, $^{\$/}{*}p<0.05$, $^{\$\$/}{**}p<0.01$, $^{\$\$\$/}{***}p<0.001$. N=3, One-way ANOVA followed by Tukey's multiple comparison.

Supplementary Fig. 9. The uncut and full blots for the gels shown in Fig. 1.

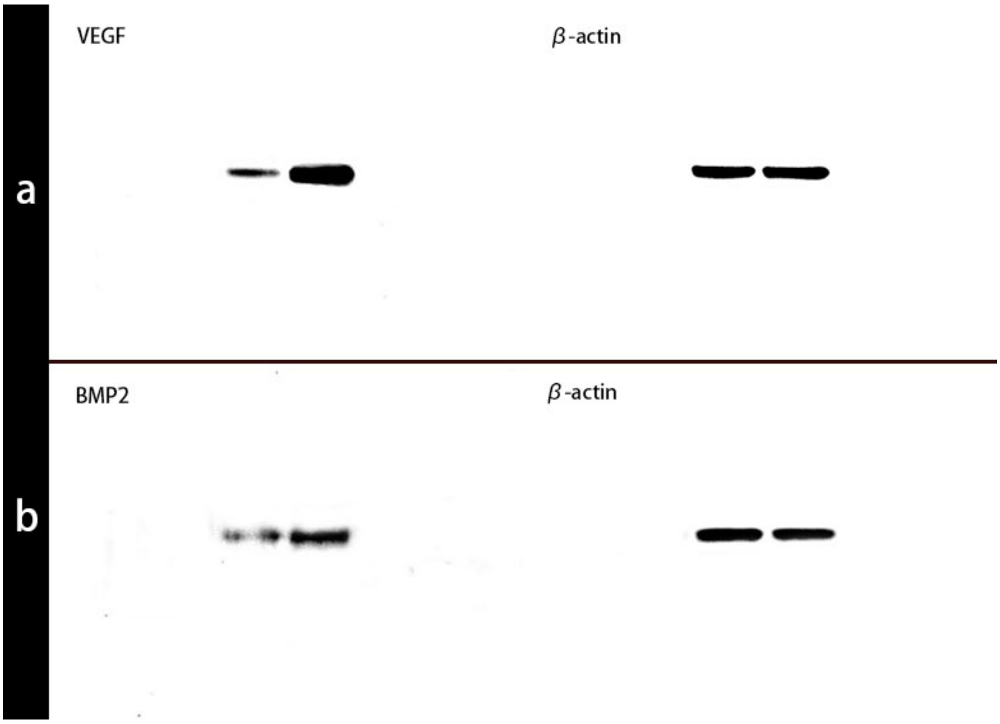

(a) The uncut and full blots for the blots shown in the Fig. 1c. (b) The uncut and full blots for the blots shown in the Fig. 1f.

Supplementary Fig. 10. The uncut and full blots for the gels shown in Fig. 2k.

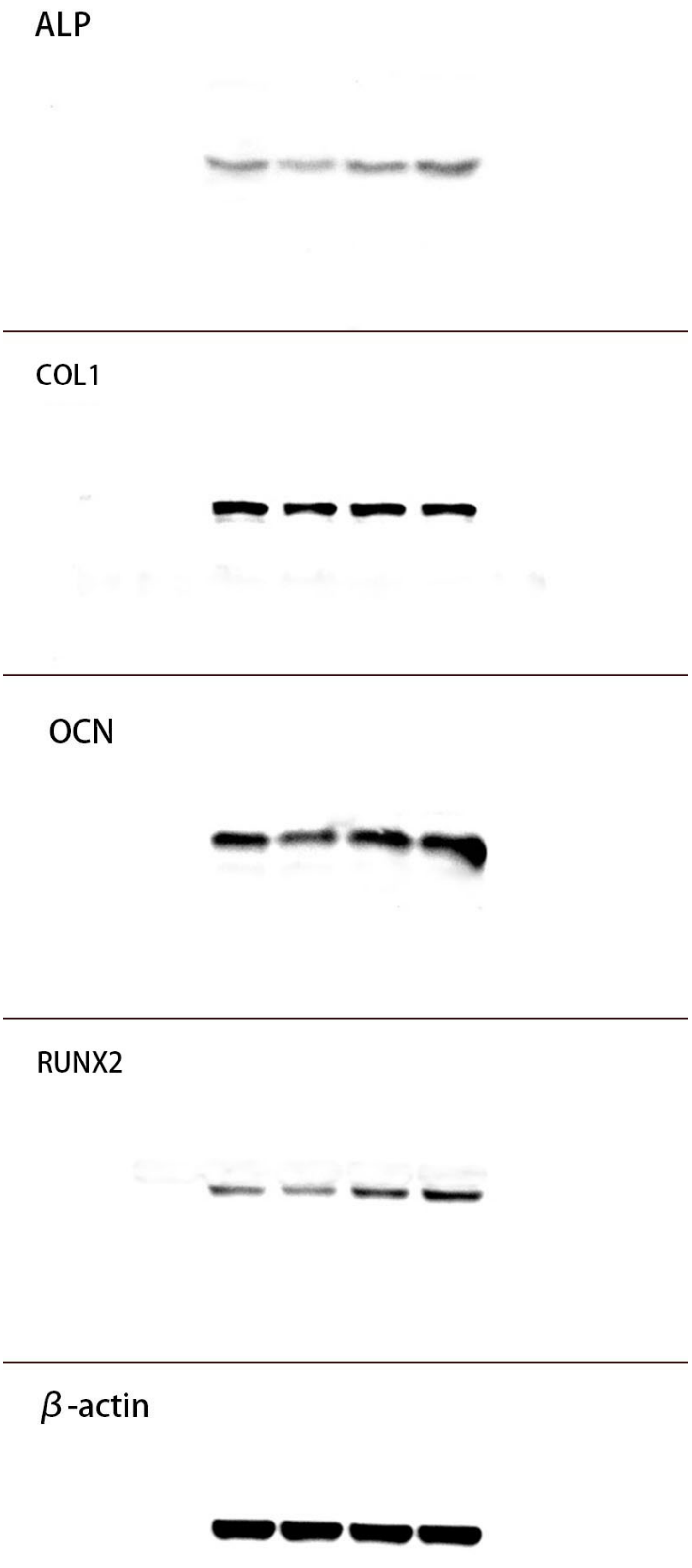

Supplementary Fig. 11. The full blots for the blots shown in Fig. 6.

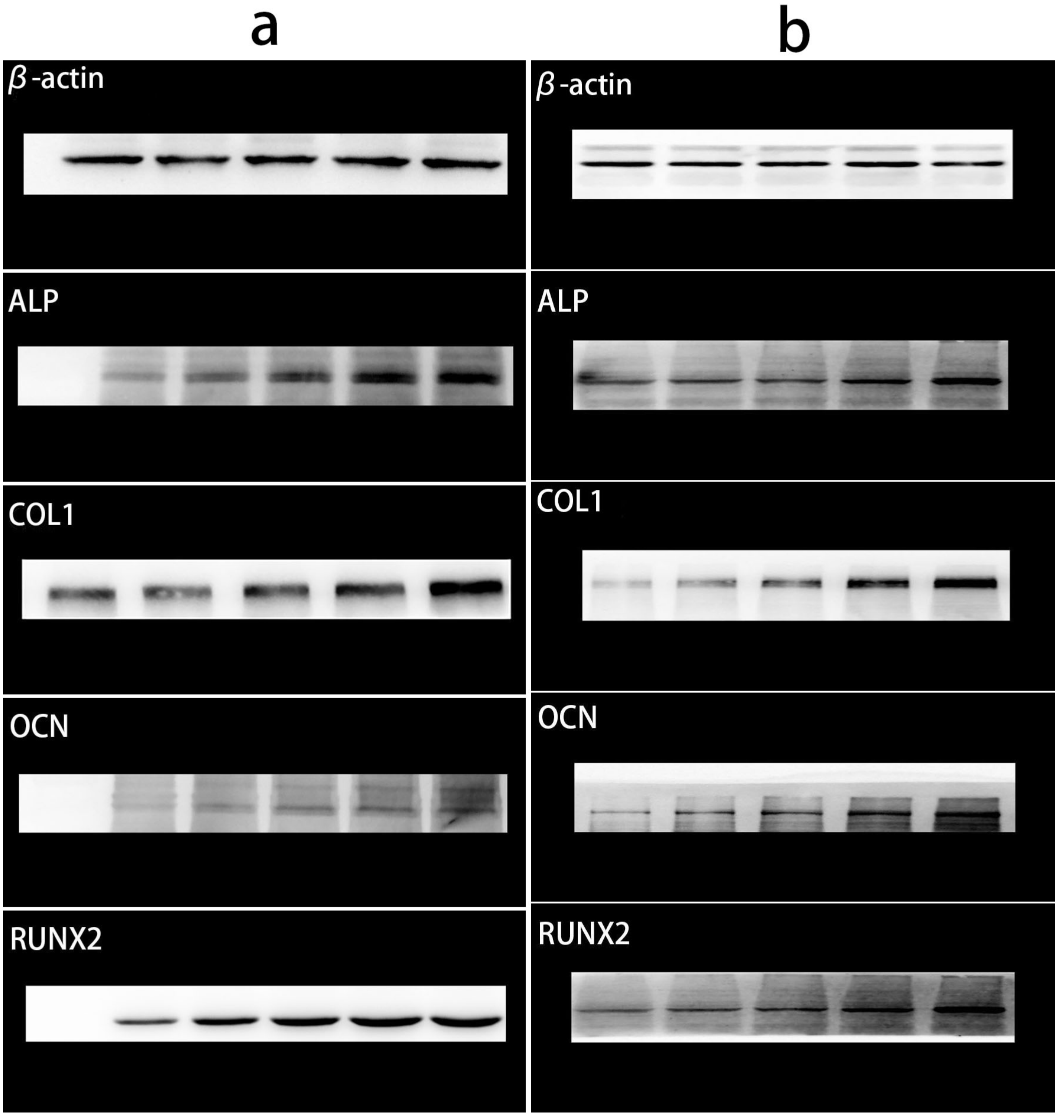

(a)The full blots for the blots shown in the Fig. 6e. (b) The full blots for the blots shown in the Fig. 6n.

Supplementary Fig. 12. The uncut and full blots for the blots shown in Supplementary Fig. 2k.

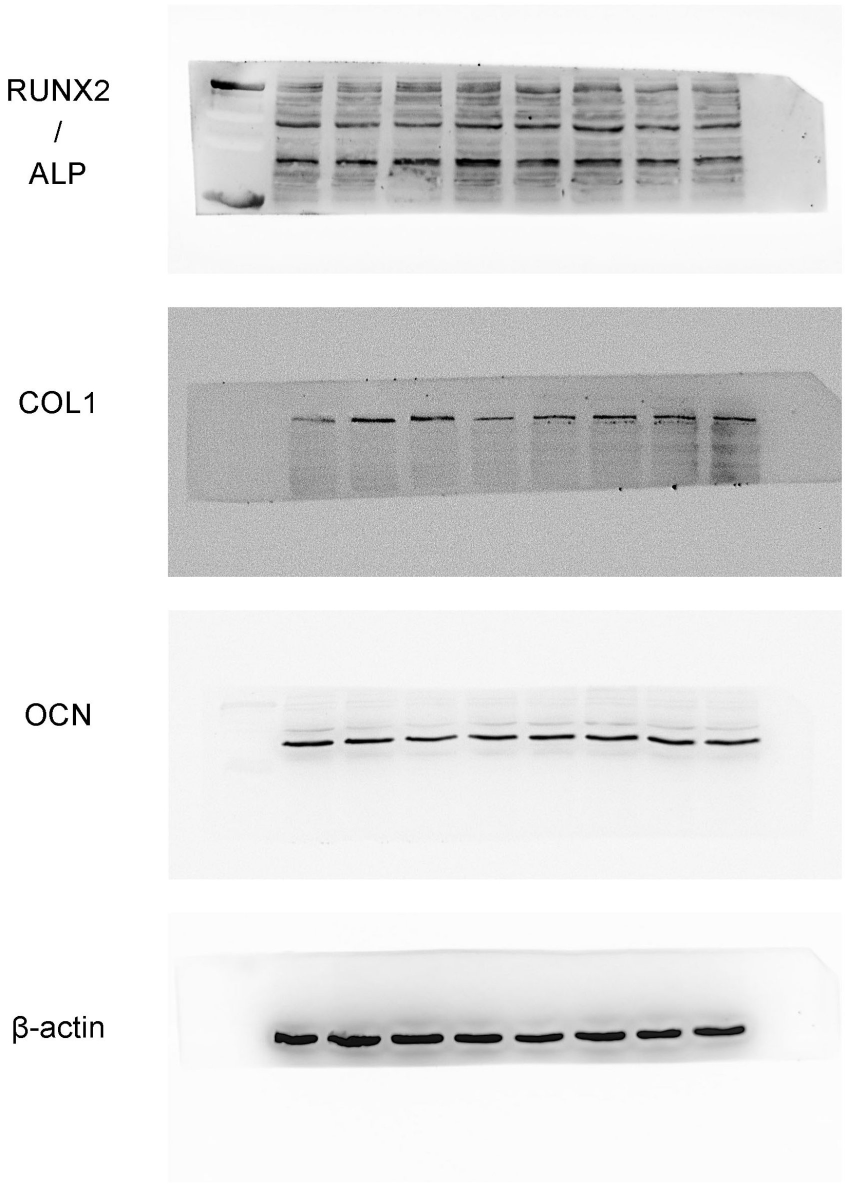

Supplementary Fig. 13. The uncut and full blots for the blots shown in Supplementary Fig. 8e.

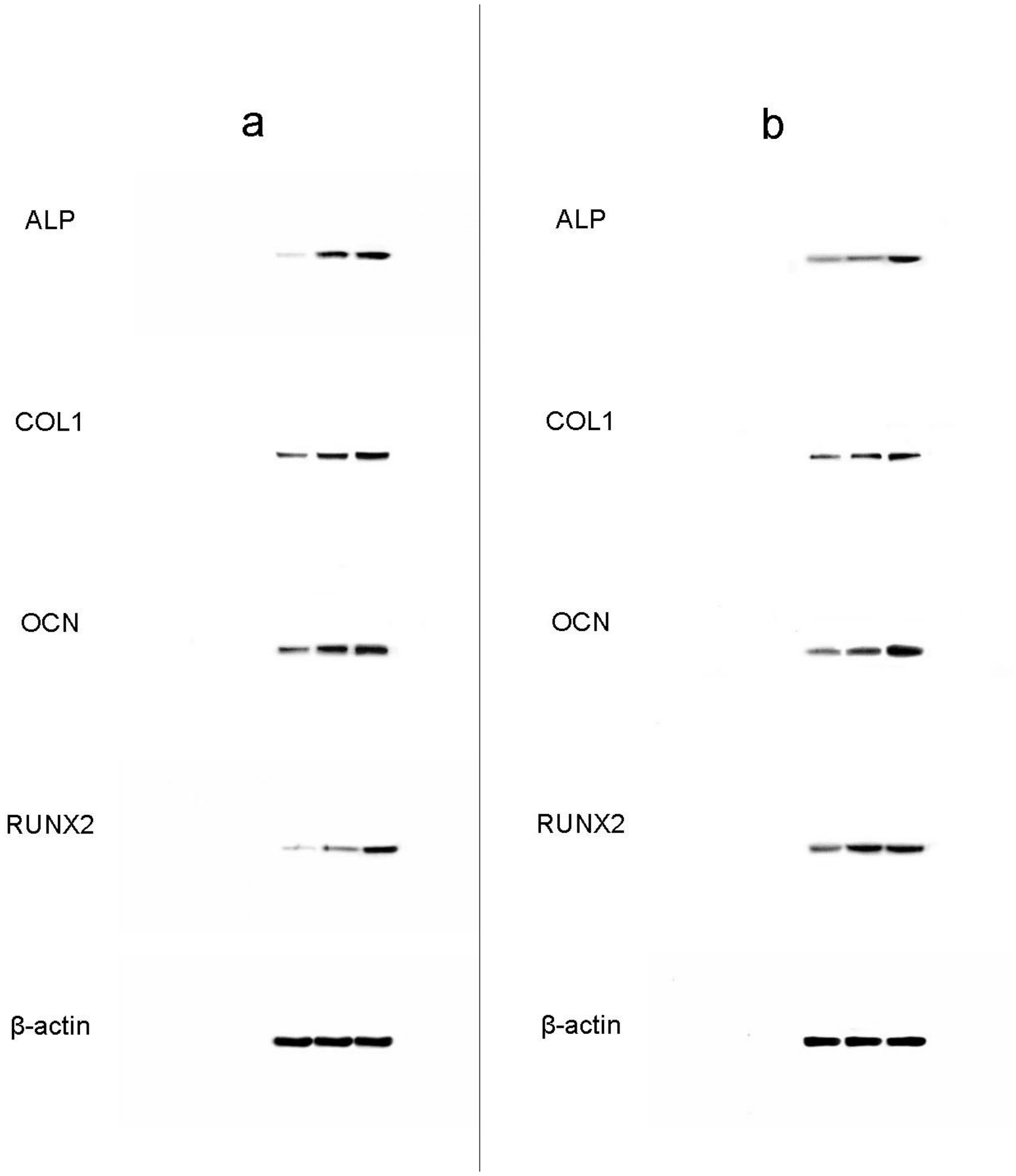

Supplementary Fig. 14. The uncut and full blots with markers for proteins ALP, COL1, OCN, RUNX2.

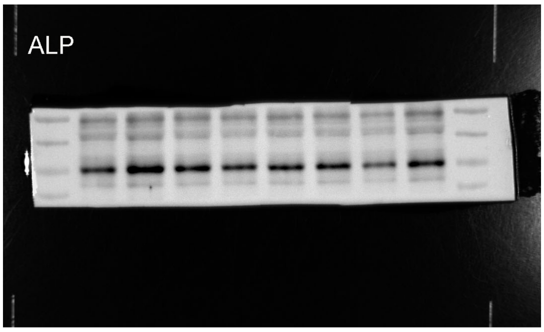

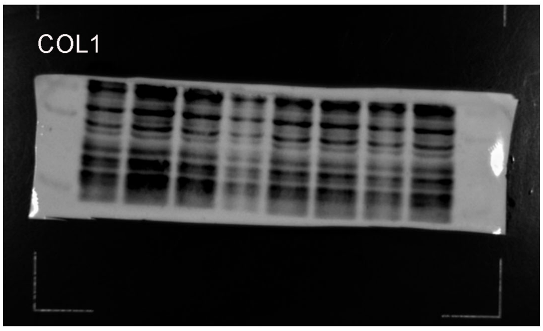

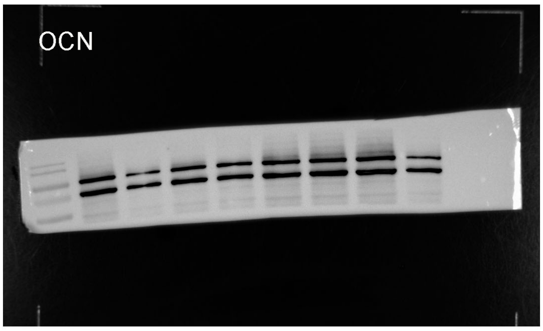

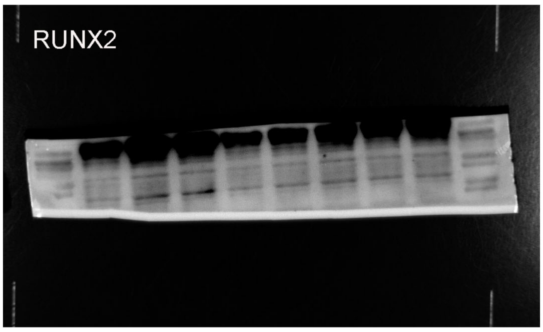

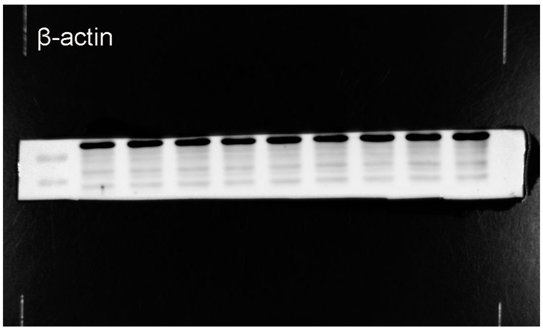

Supplementary Fig. 15. Markers of BMSCs were determined by flow cytometry.

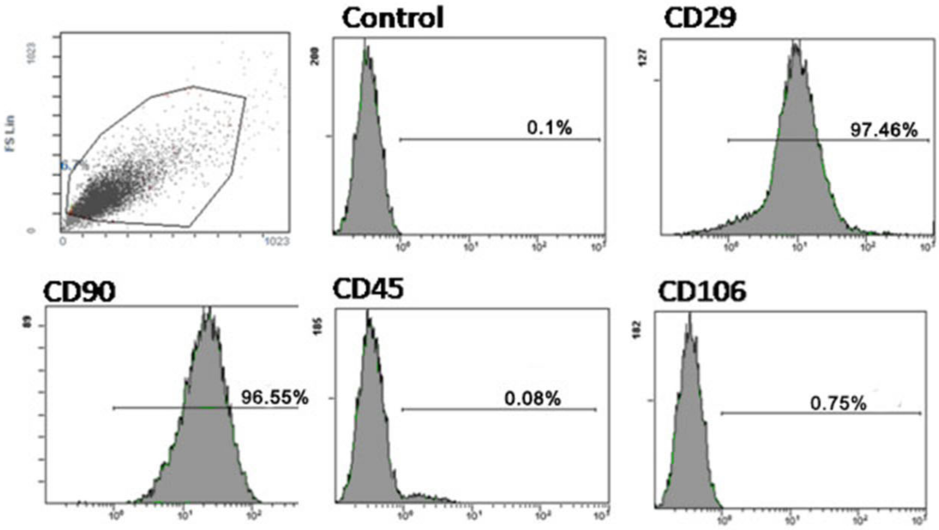

Supplement: Supplementary file 1 — Supplementary Information [file 42003_2020_1606_MOESM1_ESM.pdf]
