# Supplementary material for: BMP-2 and VEGF-A modRNAs in collagen scaffold synergistically drive bone repair through osteogenic and angiogenic pathways
Source: Commun Biol. 2021 Jan 19;4:82. doi: 10.1038/s42003-020-01606-9 (PMC7815925; doi:10.1038/s42003-020-01606-9)
Supplement: Supplementary file 2 — Description of Additional Supplementary File [file 42003_2020_1606_MOESM2_ESM.pdf]

Description of Additional Supplementary Files

File Name: Supplementary Data 1

Description: Source data file for main figures.
