# Supplementary material for: BMP-2 and VEGF-A modRNAs in collagen scaffold synergistically drive bone repair through osteogenic and angiogenic pathways
Source: Commun Biol. 2021 Jan 19;4:82. doi: 10.1038/s42003-020-01606-9 (PMC7815925; doi:10.1038/s42003-020-01606-9)
Supplement: Supplementary file 4 — Reporting Summary [file 42003_2020_1606_MOESM4_ESM.pdf]

nature research

Corresponding author(s): Jie Yuan,Kenneth Chien,MinWei,Wei Fu

Last updated by author(s): Dec 10, 2020

Reporting Summary

Nature Research wishes to improve the reproducibility of the work that we publish. This form provides structure for consistency and transparency in reporting. For further information on Nature Research policies, see Authors & Referees and the Editorial Policy Checklist.

Statistics

For all statistical analyses, confirm that the following items are present in the figure legend, table legend, main text, or Methods section.

n/a	Confirmed	
☐	☒	The exact sample size (*n*) for each experimental group/condition, given as a discrete number and unit of measurement
☐	☒	A statement on whether measurements were taken from distinct samples or whether the same sample was measured repeatedly
☐	☒	The statistical test(s) used AND whether they are one- or two-sided<br>*Only common tests should be described solely by name; describe more complex techniques in the Methods section.*
☐	☒	A description of all covariates tested
☐	☒	A description of any assumptions or corrections, such as tests of normality and adjustment for multiple comparisons
☐	☒	A full description of the statistical parameters including central tendency (e.g. means) or other basic estimates (e.g. regression coefficient) AND variation (e.g. standard deviation) or associated estimates of uncertainty (e.g. confidence intervals)
☐	☒	For null hypothesis testing, the test statistic (e.g. *F*, *t*, *r*) with confidence intervals, effect sizes, degrees of freedom and *P* value noted<br>*Give P values as exact values whenever suitable.*
☐	☒	For Bayesian analysis, information on the choice of priors and Markov chain Monte Carlo settings
☐	☒	For hierarchical and complex designs, identification of the appropriate level for tests and full reporting of outcomes
☐	☒	Estimates of effect sizes (e.g. Cohen's *d*, Pearson's *r*), indicating how they were calculated

Our web collection on statistics for biologists contains articles on many of the points above.

Software and code

Policy information about availability of computer code

Data collection	Microsoft
Data analysis	GraphPad Prism Version 7

For manuscripts utilizing custom algorithms or software that are central to the research but not yet described in published literature, software must be made available to editors/reviewers. We strongly encourage code deposition in a community repository (e.g. GitHub). See the Nature Research guidelines for submitting code & software for further information.

Data

Policy information about availability of data

All manuscripts must include a data availability statement. This statement should provide the following information, where applicable:

- Accession codes, unique identifiers, or web links for publicly available datasets
- A list of figures that have associated raw data
- A description of any restrictions on data availability

The authors declare that [the/all other] data supporting the findings of this study are available within the paper [and its supplementary information].

Field-specific reporting

Please select the one below that is the best fit for your research. If you are not sure, read the appropriate sections before making your selection.

☒ Life sciences ☐ Behavioural & social sciences ☐ Ecological, evolutionary & environmental sciences

For a reference copy of the document with all sections, see nature.com/documents/nr-reporting-summary-flat.pdf

Life sciences study design

All studies must disclose on these points even when the disclosure is negative.

Sample size	Animal experiments were performed at n=6, in vitro experiments were performed with n=3.
Data exclusions	no data were excluded from the analysis
Replication	all attempts were performed twice with n=3. (2x (n=3)=6)
Randomization	samples were allocated into experimental groups randomly
Blinding	blinding was not relevant to our study because our research was about animal experiments

Reporting for specific materials, systems and methods

We require information from authors about some types of materials, experimental systems and methods used in many studies. Here, indicate whether each material, system or method listed is relevant to your study. If you are not sure if a list item applies to your research, read the appropriate section before selecting a response.

Materials & experimental systems

n/a	Involved in the study	
☐	☒	Antibodies
☒	☐	Eukaryotic cell lines
☒	☐	Palaeontology
☐	☒	Animals and other organisms
☒	☐	Human research participants
☒	☐	Clinical data

Methods

n/a	Involved in the study	
☒	☐	ChIP-seq
☐	☒	Flow cytometry
☒	☐	MRI-based neuroimaging

Antibodies

Antibodies used	For in vitro experiments, the primary antibodies including rabbit anti-ALP, rabbit anti-COL1, rabbit anti-OCN, and rabbit anti-RUNX2, rabbit anti-VEGF,rabbit anti-BMP-2 were used in western blot experiments. These antibodies were all purchased from abcam, with the following catalogue numbers: Ab95462, Ab34710, Ab93876, Ab192256,ab46154, ab214821 respectively. The secondary antibody HRP-conjugated goat anti-rabbit IgG was from Beyotime, with catalog number 111-035-045.<br>For ex vivo studies, we used the following primary antibodies: rabbit anti-ALP, rabbit anti-COL1, rabbit anti-OCN, and rabbit anti-RUNX2 which were applied for western blot experiments and were purchasedfrom abcam. These antibodies are associated with the following catalogue numbers: Ab83259, Ab34710, Ab93876, Ab192256, respectively. The secondary antibody HRP-conjugated goat anti-rabbit IgG was from Beyotime, with catalog number A0208.
Validation	Each primary antibody for the species and application was validated. We performed 2ndary antibody alone staining to ensure the specifity of the Abs.

Animals and other organisms

Policy information about studies involving animals; ARRIVE guidelines recommended for reporting animal research

Laboratory animals	8-week-old male Spraguee Dawley rats
Wild animals	*Provide details on animals observed in or captured in the field; report species, sex and age where possible. Describe how animals were caught and transported and what happened to captive animals after the study (if killed, explain why and describe method; if released, say where and when) OR state that the study did not involve wild animals.*
Field-collected samples	Animals were housed in the animal experimental center of Shanghai ninth people's hospital (China), the temperature was about 20?.
Ethics oversight	All animal experiments were approved by the affiliated Ninth People's Hospital Institutional Animal Care and Use Committee, Shanghai Jiaotong University, School of Medicine, in advance. This is stated in our manuscript.

Note that full information on the approval of the study protocol must also be provided in the manuscript.

Flow Cytometry

Plots

Confirm that:

☒ The axis labels state the marker and fluorochrome used (e.g. CD4-FITC).

☒ The axis scales are clearly visible. Include numbers along axes only for bottom left plot of group (a 'group' is an analysis of identical markers).

☒ All plots are contour plots with outliers or pseudocolor plots.

☒ A numerical value for number of cells or percentage (with statistics) is provided.

Methodology

Sample preparation	24 hours after GFP and mCherry modRNA transfection, the cells were digested with trypsin, centrifuged and resuspended with PBS for the flow cytometry analysis. mRNA produced GFP and RFP compounds did not require antibody labeling/detection
Instrument	Epics Altka□Beckman□
Software	We used CytExpert for analysis
Cell population abundance	0.1 million cells were analyzed in each group
Gating strategy	FSC-A / SSC-A of starting material allowed us to gate between viable cells and debri. Singlet populations were gated before gating for GFP positive cells. Untransfected cells not expressing the respective reporters were used as negative controls

☒ Tick this box to confirm that a figure exemplifying the gating strategy is provided in the Supplementary Information.
